# A Systems Perspective: How Social–Ecological Networks Can Improve Our Understanding and Management of Biological Invasions

**DOI:** 10.1093/biosci/biaf174

**Published:** 2025-12-04

**Authors:** Fiona S Rickowski, Florian Ruland, Örjan Bodin, Thomas Evans, Mike S Fowler, Lotta C Kluger, Guillaume Latombe, Bernd Lenzner, Rafael L Macêdo, Tim Adriaens, Robert Arlinghaus, Gustavo A Castellanos-Galindo, Jaimie T A Dick, James W E Dickey, Franz Essl, Belinda Gallardo, Sabine Hilt, Yuval Itescu, Ivan Jarić, Sophia Kimmig, Lohith Kumar, Ana Novoa, Francisco J Oficialdegui, Cristian Pérez-Granados, Petr Pyšek, Wolfgang Rabitsch, David M Richardson, Núria Roura-Pascual, Menja von Schmalensee, Florencia A Yannelli, Montserrat Vilà, Giovanni Vimercati, Jonathan M Jeschke

**Affiliations:** Leibniz Institute of Freshwater Ecology and Inland Fisheries, Berlin, Germany; Institute of Biology, Freie Universität Berlin, Berlin, Germany; Berlin–Brandenburg Institute of Advanced Biodiversity Research, Berlin, Germany; Leibniz Institute of Freshwater Ecology and Inland Fisheries, Berlin, Germany; Institute of Biology, Freie Universität Berlin, Berlin, Germany; Berlin–Brandenburg Institute of Advanced Biodiversity Research, Berlin, Germany; West Iceland Nature Research Centre, Stykkishólmur, Iceland; Stockholm Resilience Centre, Stockholm University, Stockholm, Sweden; Institute of Biology, Freie Universität Berlin, Berlin, Germany; le laboratoire Ecologie, Systématique et Evolution, Université Paris-Saclay, Paris, France; Department of Biosciences, Faculty of Science and Engineering, Swansea University, Swansea, Wales, United Kingdom; Center for Ocean and Society, Kiel University, Kiel, Germany; Department of Agricultural Economics, Kiel University, Kiel, Germany; Institute of Ecology and Evolution, University of Edinburgh, Edinburgh, Scotland, United Kingdom; Division of BioInvasions, Global Change, and Macroecology, Department of Botany and Biodiversity Research, University of Vienna, Vienna, Austria; Division of BioInvasions, Global Change, and Macroecology, Department of Botany and Biodiversity Research, University of Vienna, Vienna, Austria; Leibniz Institute of Freshwater Ecology and Inland Fisheries, Berlin, Germany; Institute of Biology, Freie Universität Berlin, Berlin, Germany; Research Institute for Nature and Forest, Brussels, Belgium; Leibniz Institute of Freshwater Ecology and Inland Fisheries, Berlin, Germany; Division of Integrative Fisheries Management, Faculty of Life Sciences at Humboldt-Universität zu Berlin, Berlin, Germany; Leibniz Institute of Freshwater Ecology and Inland Fisheries, Berlin, Germany; Institute of Biology, Freie Universität Berlin, Berlin, Germany; Smithsonian Tropical Research Institute, Balboa, Panama; Institute for Global Food Security, School of Biological Sciences, Queen’s University Belfast, Belfast, Northern Ireland, United Kingdom; Leibniz Institute of Freshwater Ecology and Inland Fisheries, Berlin, Germany; Institute of Biology, Freie Universität Berlin, Berlin, Germany; Berlin–Brandenburg Institute of Advanced Biodiversity Research, Berlin, Germany; GEOMAR Helmholtz Centre for Ocean Research Kiel, Kiel, Germany; National Laboratory for Health Security, HUN-REN Centre for Ecological Research, Budapest, Hungary; Division of BioInvasions, Global Change, and Macroecology, Department of Botany and Biodiversity Research, University of Vienna, Vienna, Austria; Instituto Pirenaico de Ecología, Spanish National Research Council, Zaragoza, Spain; Leibniz Institute of Freshwater Ecology and Inland Fisheries, Berlin, Germany; Leibniz Institute of Freshwater Ecology and Inland Fisheries, Berlin, Germany; Institute of Biology, Freie Universität Berlin, Berlin, Germany; Department of Evolutionary and Environmental Biology, University of Haifa, Haifa, Israel; Université Paris-Saclay, CNRS, AgroParisTech, Ecologie Systématique Evolution, Gif-sur-Yvette, France; Biology Centre of the Czech Academy of Sciences, Institute of Hydrobiology, České Budějovice, Czech Republic; Leibniz Institute of Freshwater Ecology and Inland Fisheries, Berlin, Germany; Institute of Biology, Freie Universität Berlin, Berlin, Germany; Leibniz Institute of Freshwater Ecology and Inland Fisheries, Berlin, Germany; Institute of Biology, Freie Universität Berlin, Berlin, Germany; ICAR-Central Inland Fisheries Research Institute, Barrackpore, India; Department of Invasion Ecology, Institute of Botany, Czech Academy of Sciences, Průhonice, Czech Republic; Estación Experimental de Zonas Áridas, Consejo Superior de Investigaciones Científicas, Almería, Spain; Department of Invasion Ecology, Institute of Botany, Czech Academy of Sciences, Pruhonice, Czech Republic; Department of Conservation Biology and Global Change, Doñana Biological Station (CSIC), Seville, Spain; Faculty of Fisheries and Protection of Waters, South Bohemian Research Centre of Aquaculture and Biodiversity of Hydrocenoses, University of South Bohemia in České Budějovice, České Budějovice, Czech Republic; Biodiversity Conservation and Management Programme, Forest Science and Technology Center of Catalonia (CTFC), Solsona, Catalonia, Spain; Department of Invasion Ecology, Institute of Botany, Czech Academy of Sciences, Průhonice, Czech Republic; Institute of Botany, Czech Academy of Sciences, Průhonice, Department of Ecology, Faculty of Science, Charles University, Prague, Czech Republic; Environment Agency Austria, Vienna, Austria; Department of Invasion Ecology, Institute of Botany, Czech Academy of Sciences, Průhonice, Czech Republic; Centre for Invasion Biology, Department of Botany and Zoology, Stellenbosch University, Stellenbosch, South Africa; Departament de Ciències Ambientals, Facultat de Ciències, Universitat de Girona, in Girona, Catalonia, Spain; West Iceland Nature Research Centre, Stykkishólmur, Iceland; Leibniz Institute of Freshwater Ecology and Inland Fisheries, Berlin, Germany; Institute of Biology, Freie Universität Berlin, Berlin, Germany; Argentine Institute for Dryland Research, CONICET and the Universidad Nacional de Cuyo, Mendoza, Argentina; Estación Biológica de Doñana, with the Department of Plant Biology and Ecology, Universidad de Sevilla, in Sevilla, Spain; Department of Biology, University of Fribourg, Fribourg, Switzerland; Leibniz Institute of Freshwater Ecology and Inland Fisheries, Berlin, Germany; Institute of Biology, Freie Universität Berlin, Berlin, Germany; Berlin–Brandenburg Institute of Advanced Biodiversity Research, Berlin, Germany

**Keywords:** impacts of nonnative species, invasive alien species, management of biological invasions, social–ecological networks, social–ecological system

## Abstract

Reversing biodiversity loss and the sustainability crisis requires approaches that explicitly consider human–nature interdependencies. Social–ecological networks, which incorporate social and ecological actors and entities, as well as their interactions, provide such an approach. Social–ecological networks have been applied to a range of complex issues, including sustainable resource use, management of ecosystem services and disservices, and collective action. However, the application of social–ecological networks to invasion science remains limited so far, despite their clear potential for studying human contributions to introduction pathways of nonnative species, invasion success, direct and indirect impacts, and their management. In the present article, we review past applications of social–ecological networks to biological invasions, provide guidance on how to construct and analyze such networks, with an illustrative example, and outline future opportunities of social–ecological networks in invasion science. We aim to inform and inspire the applications of social–ecological networks to improve our ability to meet the diverse challenges facing invasion science.

Anthropogenic impacts on biodiversity, such as species extinctions or functional degradation, also include those resulting from the intentional and unintentional transport of species to regions where they would not naturally occur; such species are termed *nonnative* or *alien*. A subset of these species may become invasive if they spread beyond the places where they have been introduced and have negative or deleterious impacts on native biodiversity (Roy et al. 2023). Invasive species are recognized as driving forces of the ongoing global biodiversity loss (IPBES 2019, Roy et al. 2023, Turbelin et al. 2023). Their impacts on native species can be devastating, both directly (e.g., through predation, parasitism, or hybridization) and indirectly (e.g., by transmitting pathogens and disrupting well-established predator–prey interactions) (Vilà et al. 2011, Blackburn et al. 2014, Linders et al. 2019, Kumschick et al. 2020). They may also cause ecosystem-scale changes—for example, through the alteration of community composition, trophic cascades, or ecosystem engineering (Pyšek et al. 2020, Roy et al. 2023, Bacher et al. 2024). In addition, invasive species lead to substantial financial costs through damage and management, affecting many economic sectors (Diagne et al. 2020, Novoa et al. 2021). They affect human health and well-being (Mazza and Tricarico 2018), as they can spread diseases (Zhang et al. 2022), cause allergies (Bernard-Verdier et al. 2022), be venomous or toxic (Nentwig et al. 2017), and disrupt recreational activities and other social and cultural practices (Pyšek et al. 2020, Bacher et al. 2024). However, not all nonnative species are invasive, and both invasive and noninvasive nonnative species can have positive or beneficial ecological or socioeconomic effects (Vimercati et al. 2020). For example, nonnative species can fulfil the functional role of a (locally) extinct native species (Vizentin-Bugoni et al. 2019), provide ecosystem services such as improving water quality (Neves et al. 2020, Reynolds and Aldridge 2021), or stabilize fisheries revenues (Van Rijn et al. 2020). Due to myriad concurrent anthropogenic impacts, the prioritization and choice of conservation efforts demand a holistic understanding contingent on environmental and social contexts, as well as different geographic scales (Corlett 2015, Bellard et al. 2022).

Several tools have been developed to assess the impacts of invasive species with standardized and evidence-based approaches (for an overview, see González-Moreno et al. 2019, Vilà et al. 2019, Carneiro et al. 2025). For example, the International Union for Conservation of Nature (IUCN) Environmental Impact Classification for Alien Taxa (EICAT) is a protocol for assessing deleterious ecological impacts of nonnative species on native biodiversity (Blackburn et al. 2014, IUCN 2020). Similarly, the EICAT+ protocol guides assessments of beneficial ecological impacts (Vimercati et al. 2022), whereas the SEICAT and SEICAT+ protocols are focused on deleterious and beneficial socioeconomic impacts on human well-being respectively (the *S* is for *socioeconomic*; Bacher et al. 2018, Vimercati et al. 2025, https://doi.org/10.32942/X28M09 [preprint: not peer reviewed]). Other approaches to assess nonnative species impacts have been developed, including the estimation of monetary costs (InvaCost; Diagne et al. 2020), the use of functional and numerical response parameters in consumer–resource interactions (Dick et al. 2014, Dickey et al. 2020), or the dispersal–origin–status–impact framework, which integrates dispersal mechanisms, species origin, and population status while addressing a range of impacts such as ecological, economic, cultural, or health-related (Soto et al. 2024). Assessments of the future risks associated with biological invasions include horizon-scanning techniques (Verbrugge et al. 2010, Srėbalienė et al. 2019). However, none of these approaches capture how different types of impacts are interrelated (Leung et al. 2012).

A broad understanding of the full range of nonnative species impacts, synergies, and conflicts is important to make informed management decisions (Vilà and Hulme 2017, Stevenson et al. 2023, Roura‐Pascual et al. 2024). Deciding which of the many existing management options to apply (Robertson et al. 2020, Roy et al. 2023) requires weighing their social and ecological costs and benefits in a given context. Invasive species and their impacts can be negatively perceived by some stakeholders but positively by others, and that perception may shift over time and space (Simberloff et al. 2013, Cottet et al. 2015). For example, fish species such as rainbow trout (*Oncorhynchus mykiss*) or brown trout (*Salmo trutta*) have been introduced to many ecosystems to increase the recreational value for anglers and for aquaculture purposes, but they have negatively affected native taxa that can, in turn, be important to other fisheries (Jeschke et al. 2022). Likewise, nuisance caused by invasive aquatic macrophytes may be perceived as more problematic by residents than by visitors (Thiemer et al. 2023). Invasive trees can be aesthetically pleasing (Vaz et al. 2018) while simultaneously eliminating suitable habitat for native insects (Litt et al. 2024), birds (Grzędzicka and Reif 2020), or plants (Sádlo et al. 2017) or radically altering ecosystem services (van Wilgen et al. 2022, Romero-Blanco et al. 2023). Similarly, an environmental nongovernmental organization might favor the eradication of an invasive plant, aiming to reduce its impacts on native flora, whereas local farmers would rather plant it to increase the soil quality (Benediktsson 2015, Lojeski and Plante 2021). Incorporating active stakeholder engagement, such as participatory workshops or citizen science initiatives, is vital for developing effective management strategies by fostering collaborative knowledge production and integrating diverse perspectives into decision-making (Novoa et al. 2018, Shackleton et al. 2019, Nuñez et al. 2022). As invasion management is an adaptive process requiring a governance structure, legal framework, and typically public support, it is crucial to study biological invasions as part of a social–ecological system (Richardson 2010, Frost et al. 2019, Hui and Richardson 2019, Groom et al. 2021, Heger et al. 2021).

Social–ecological systems are complex adaptive systems comprising humans and nature, as well as their relationships (IPBES 2019). They are dynamic and open (i.e., they change in reaction to external drivers through time), as well as being context dependent and producing emergent phenomena (i.e., characteristics that exist because of the interplay of the system components; Preiser et al. 2021). Social–ecological networks—networks mapping interactions between humans and nature (see the glossary in supplement S1)—are tools used to understand relations (i.e., interactions) between entities. They complement social–ecological systems frameworks (e.g., common pool resource governance; Ostrom 2009), which take a more qualitative approach, and system dynamic models (e.g., Stella, iseesystems.com), which model causal relationships between variables. Social–ecological networks can incorporate both qualitative and quantitative data in a structured way. They can disentangle direct and indirect connectivity and interdependencies between human–nature interfaces and can inform management initiatives at multiple scales (Bodin 2017, Beever et al. 2019, Kluger et al. 2019, Sayles et al. 2019, Kluger et al. 2020, Felipe-Lucia et al. 2022). Social–ecological networks have been applied in the context of biological invasions (table 1) and, for example, have identified management actions required to ensure a functioning ecosystem (e.g., Ortiz et al. 2015).

**Table 1.** Published studies of social–ecological networks that incorporate biological invasions (for an overview of the search protocol and inclusion criteria, see supplement S2).

Study	Research focus	Relevant themes in invasion science	Network type	Nodes (E, S) and links (EE, SS, SE)	social–ecological network articulation	Invasive species	Data source(s)	Network analysis	Key findings in brief
Alexander et al. (2017)^s,u^	Governance networks in marine protected areas (MPAs); Social–ecological fit	Management, spread	Multilevel, directed	E: MPAs S: governing organizations EE: ecological connectivity SS: information SS: management SS: collaboration SE: management strategies	Type III	*Pterois miles* and *Pterois volitans* as node attribute	Sociometric survey, semistructured interviews, legal documents	In degree centrality, betweenness centrality, density.	Multilevel vertical ties (local–national) enhanced fit, addressing functional misfits (e.g., invasive lionfish removal).
Haak et al. (2017)^s,u^	Angler movement data with ecosystem models to evaluate the risks and impacts of species invasion	Pathways, impact, management	Directed, weighted, nested	E: species E: lakes or reservoirs EE: trophic interactions SS: angler movement SE: fishing	Type II	*Bellamya chinensis* as node, node and link attributes	Angler interviews	Contagion models (angler movement) Ecopath models (ecosystem trophic interactions)	Expanded on management implications, network structure, and ecosystem-level effects of the invasive species (*Bellamya chinensis*).
Fried et al. (2022)^s^	Fit between environmental governance and biophysical systems	Management	Bipartite, directed, weighted	E: water quality, invasive species S: managing actors EE: issue interdependencies SE: management actions by actors to address issues	Type II	Unspecified node	Text analysis, internet search	Bipartite exponential random graph models	Addressing environmental problems holistically—by considering their interdependencies and leveraging specialized or regional actors—leads to better outcomes for governance and sustainability.
Contesse et al. (2021)^s^	Nonhuman agency	Impact, management	Ego network	E: *Bagrada hilaris* S: stakeholders SE: interactions	Type II	*Bagrada hilaris* as node	Interviews	Qualitative	The study highlights how *Bagrada hilaris* acts as a nonhuman agent, catalyzing sustainability transitions by reshaping pest management networks.
Sinclair et al. (2021)^s^	Introduction pathways via the pet trade, with attention to species transport dynamics and risk factors	Pathways	Directed network	S: actors and entities involved in pet trade SE: relationships and transactions in the pet trade (e.g., trade volumes, regulatory interactions)	Type I	Unspecific, vertebrate pet trade (fish, amphibians and reptiles) as links	Scientific literature, databases	Qualitative	This study highlights the importance of regulating pet trade pathways to mitigate invasion risks and emphasizes the interconnected roles of stakeholders in facilitating or preventing introductions.
Ashander et al. (2022)^s^	Large-scale management of invasive species, control strategies, ecological information	Management, Pathways	Directed, weighted	E: lakes SS: boater movements	Type I	*Dreissena polymorpha* as node and link attributes	Boater movement data (2014–2017), zebra mussel infestation status	Metrics Degree, H + A, Betweenness centrality, ILP optimization	Network-based management using Degree and H + A centrality achieved near-optimal performance, especially under constrained budgets.
Drake et al. (2010)^s^	Modeling vector movement, angler activity	Pathways, spread	Directed, weighted	E: angler origins or destinations (lakes) SS: movements via road network	Type I	*Dreissena polymorpha* and *Neogobius melanostomus* as vectors (angler movements)	Surveys, road networks, lake attributes	Negative binomial and zero-inflated spatial interaction models; least-cost routing; distance-decay models, generalized linear model	Least-cost routing outperforms Euclidean models in explaining vector movements. Lake size and sportfish richness strongly influence destination attractiveness.
Escobar et al. (2019)^u^	Lake connectivity and risk analysis	Pathways, spread, management	Directed, weighted	E: lakes SS: angler movement SE: watercraft movement	Type I	*Nitellopsis obtusa* as link attribute (vector as proxy)	Survey data from Lake Koronis (2013–2014)	In degree scores	Identified “super receiver” lakes, like Rice Lake, based on watercraft flow; high connectivity increases invasion risk and proposed network guided management.
Jentsch et al. (2020)^s,u^	Effectiveness of social incentives, direct interventions, and quarantines to mitigate the spread of invasive species	Pathways, management	directed	E: campgrounds (with tree attributes) SS: camper movement	Type I	*Agrilus planipennis, Anoplophora glabripennis* as attributes in pest spread model	Campground reservation data	Pest spread model (mechanistic metapopulation model)	Social incentives (e.g., reducing firewood transport) are effective at slowing spread locally but less so for reducing total infestation.
Letschert et al. (2021)^u^	Risk assessment of invasive species dispersal through ship traffic in the GMR, biofouling	Spread, management	Directed, weighted	E: anchorages at port locations SS: ship routes	Type I	*Bugula neritina, Watersipora subtorquata* as link attribute (vector of fouling species as proxy)	Port and tourism data from Galapagos National Park (DPNG), ship movement from MarineTraffic website	Based on ship routes, WSA, and vessel types; dispersal model calculated cumulative DS	Identified highly connected hubs (e.g., Port Santa Cruz and Port Baltra) as key dispersal nodes; recreational and passenger vessels play dominant roles in nonnative species spread.
Lubell et al. (2017)^s,u^	Management and governance of invasive species, stakeholder cooperation, trust	Management	Undirected	S: stakeholders SS: communication	Type I	*Spartina* sp. (hybrid population) as topic of network, not explicit	Survey, interviews	Centrality metrics, core–periphery analysis	Effective governance relies on coordinated research, consensus building, and balancing conservation trade-offs.
McAllister et al. (2015)^s^	Management pest species and disease outbreak, governance	Management	Bipartite and multilevel	S: individuals, S: groups SS: communication SS: participation	Type I	*Mycosphaerella fijiensis* as topic of network	Surveys, interviews, reconstructed response network	Exponential random graph models	Local coordination drove success, but cross-scale interactions were limited, highlighting the reliance on informal networks.
Nourani et al. (2018)^u^	Management, adaptive comanagement, social learning	Management	Directed	S: task force members (state, county or municipal staff, arborists, citizens, etc.) S: cooperation or information	Type I	Emerald Ash Borer (EAB) as topic of network	Learning assessments (cognitive, normative, relational), network surveys, interviews, document analysis	In or out degree centrality, relational learning	Task forces improved learning and collaboration, with outcomes varying by local ecological and social contexts.
Omondiagbe et al. (2017)^u^	Management of invasive species, collective action	Management	Directed	S: conservation groups S: government S: stakeholders SS: communication	Type I	*Rattus* *exulans, R. norvegicus*, *Mus musculus, Macropus* spp, *Erinaceus europaeus, Oryctolagus cuniculus, Felis catus* as topic of network	Survey	Organizational network analysis (ONA), centrality, density metrics	Conservation and ISM activities were networked but sparse; strong influence by central stakeholders.
Rebaudo et al. (2011)^s^	Modeling and educational intervention on invasive pest management	Management	Pest spread dynamics (based on ABM)	S: villages, farmers SS: human movement SE: pest dispersal between villages via farmers	Type I	*Tecia solanivora* as link attribute (human-mediated long-distance dispersal as proxy)	Scientific studies, Simulation, GIS, participatory surveys	Agent-based model	Farmers’ movements and pest control knowledge significantly influence pest spread speed.
Wyckhuys et al. (2018)^s,u^	Biological control of invasive pests	Pathways, management	Dynamic, directed, weighted	S: countries SS: trade in Casava	Type I	*Phenacoccus manihoti* and *Anagyrus lopezi* as link attribute (vector as proxy)	Field surveys, trade data	Dynamic network analysis	Effective biological control using *A. lopezi* mitigated *P. manihoti* impacts on cassava production in SE Asia.
Martínez-Sastre et al. (2020)^s^	Farmers’ perceptions and knowledge of natural enemies for biological control	Management	Directed, weighted	E: nonhuman species EE: trophic interactions SS: Perceptions of trophic interactions among natural enemies	Type I	*Cydia pomonella* as node	Surveys, questionnaires	Weighted degree and betweenness via Gephi and NodeXL, Spearmans rank correlation (rho)	Farmers valued natural enemies more for croplands in general than for cider-apple orchards. Education and farming experience influenced perceptions.
Ortiz et al. (2015)^s,u^	Management and control strategies for lionfish invasion	Management	Signed, directed	E: species, functional groups S: fishers EE: trophic interactions	Type I	*P. volitans* as node (predator and competitor)	Literature review, modeling	Qualitative loop analysis Stability criteria via Routh-Hurwitz and Levins’ criteria	Coral restoration programs enhance ecosystem stability. Harvesting lionfish is sustainable if groupers (natural predators) are not exploited.

*Note:*  ^s^ and ^u^ indicate whether the study was found with a systematic or an unsystematic search. In the column “Research focus,” studies highlighted in yellow focus on biological invasions as opposed to studies that focus on other topics but happen to include biological invasions. The column “Relevant themes in invasion science” indicates which general themes are addressed by each study, based on Musseau and colleagues (2024): pathways; invasion success, including spread, and invasibility; impact; or management. In the column “Nodes and links,” studies highlighted in purple indicate a focus on governance networks, and those in blue on spatially explicit networks of invasive species spread; also in this column, E and S refer to ecological and social nodes, respectively; therefore, EE are ecological links, SS are social links, and SE are social–ecological links. In the column “social–ecological network articulation” (*sensu* Kluger et al. 2020), type I refers to networks with only one type of link (either EE or SS), type II to networks with two types of links (either EE + SE or SS + SE), and type III to networks with all three types of links (SS, EE, and SE).

However, there is a lack of guidance on how to apply social–ecological networks in a standardized manner to enhance our understanding of biological invasions and to advance their wider application. In the present article, we explore how social–ecological networks can clarify and synthesize the various impacts and related processes associated with nonnative species. We introduce networks and their applications, identify key aspects for constructing and analyzing social–ecological networks in an invasion context, illustrate the approach with an example utilizing S/EICAT(+) data, and discuss the most promising opportunities this approach presents to invasion science, while also mentioning its limitations. We demonstrate that social–ecological networks provide an exciting avenue for future work that allows for holistic analysis of complex interdependencies surrounding the impacts and management options of nonnative species, as well as having the potential to give new insights into key questions within the field of invasion science (Musseau et al. 2024). We hope to inspire relational systems thinking and network approaches when studying biological invasions, as well as supplying a resource to get started in this complex topic.

## A brief overview of (social–ecological) networks

Since Euler’s solution to the seven bridges of Königsberg problem (Euler 1741), graph theory (see the glossary in supplement S1), which forms the basis of structural network analysis, has evolved from the mathematical study of pairwise relations to the study of complex interactions. Network science is a prolific field, with various approaches developed across disciplines—pioneered, for example, by Moreno with sociograms (Moreno and Jennings 1938) or Hannon for the structure of ecosystems (Hannon 1973)—and with increasing technical possibilities escalating into complexity science at the turn of the century (Boccaletti et al. 2006, Barabási 2013). In its simplest form, a network (also commonly termed a *graph*) consists of nodes (alternatively termed *vertices*; see the glossary) that are connected by links (also termed *edges* or *ties*; see the glossary). Networks can be found everywhere; for example, transportation networks, such as train stations (nodes) connected by tracks (links), or the animal nervous system in which neurons (nodes) are connected through synapses (links). More abstract semantic networks show theoretical concepts (nodes) and the relations between them (links), whereas coauthorship networks show scientists (nodes) and their scientific collaborations (links). We can distinguish between networks that aim to analyze topological structures, how they came to be or what effects these have, on the basis of graph theory, and those that represent causalities or ontologies. In this article, we will refer to the former as *interaction networks*, where causal relationships are not explicitly depicted (although they can be implicitly included, e.g., in the case of food webs).

Social network analysis evolved as a discipline in the early twentieth century, to investigate the structure of relationships among individuals. It is used to understand social structures and hierarchies, information flows, influence and power dynamics, and other aspects within social systems (McLevey et al. 2024). It is an important methodology for understanding how and why humans behave the way they do and, therefore, how phenomena such as social norms, collective action, and self-organization emerge in different contexts (Bodin 2017, Teodoro et al. 2021). Social network analysis has also been applied to other animal species—for example, to study the composition and dynamics of bird groups (Silk et al. 2014), the invasibility of fish assemblages (Beyer et al. 2010), and cultural behavior of dolphins (Mann et al. 2012).

In invasion science, ecological network analysis has been applied to assess the impacts of invasive species on biotic interactions such as pollination (Vilà et al. 2009), community assembly (Strong and Leroux 2014, David et al. 2017) and for modeling the spread of nonnative species across discrete habitats (Woodford et al. 2013, Ferrari et al. 2014). The strength and frequency of interactions among network components have been shown to affect the invasion success and impacts of nonnative species, and a network’s stability (see the glossary in supplement S1) can give insights into the invasibility of a system (Frost et al. 2019, Groom et al. 2021, Hui and Richardson 2022). Stability is the ability of the system to move toward or stay close to an equilibrium (see the glossary), which is the system’s ability to recover from change (Frost et al. 2019, Biggs et al. 2021, Hui and Richardson 2022). More specifically, we can talk about demographic stability as in population numbers and structural stability as in interactions between system components, such as in a food web (Hui and Richardson 2022). If demographic stability ceases, the population will crash and die out, whereas if trophic links in a food web are lost (such as between producers and consumers), the entire system can fail to function.

**Figure 1.**
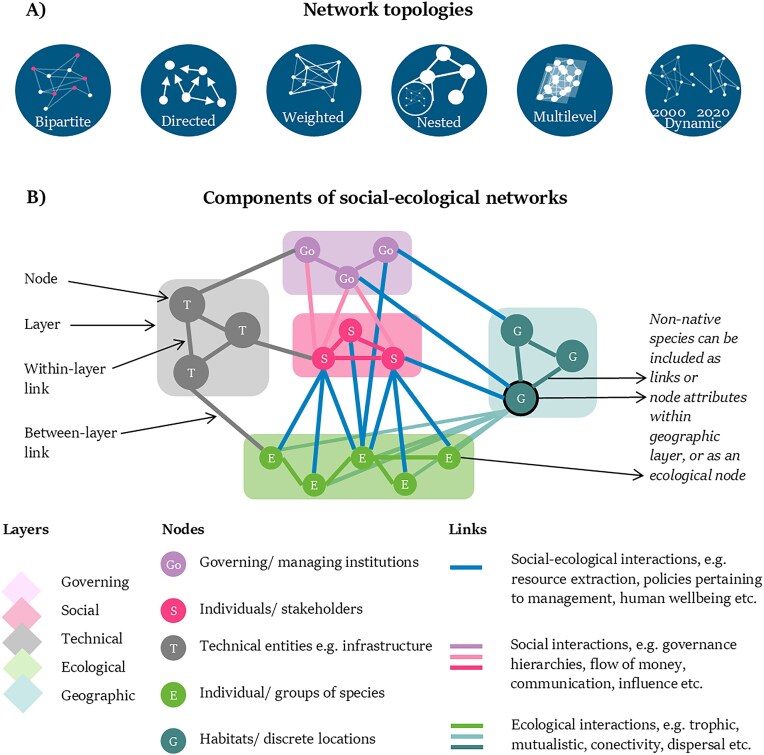
(a) Illustrations of different simplified network topologies: Bipartite: a network where links only exist between two different node types. Directed: networks where links have a direction (i.e., going from one node to another), including reciprocal relationships. Weighted: links have different strengths. Nested: networks within nodes of another network. Multilevel: multiple connected networks with a given node and link type per layer, with further links between the layers. Dynamic: networks with a temporal component (e.g., the structure of the network may change at different points across time). (b) Schematic representation of different components of social–ecological networks with example layers and nodes as well as within- and between-layer links.

Beyond these specific examples, networks have many different topologies that can be defined via their nodes, links, layers (see the glossary in supplement S1), and temporal scales (e.g., bipartite, directed, dynamic; see figure 1a). There are also specific networks from different disciplines (e.g., food webs or sociograms). Unipartite, bipartite, and multipartite networks (see the glossary) refer to the number of node types within the network. Directed (as opposed to undirected; see the glossary) networks have links coming from and going to specific nodes (e.g., food webs) and can include reciprocal links. Weighted networks (see the glossary) assign a value to the link (e.g., the amount of biomass being consumed or the number of times a pollinator visits a plant), and nested networks (see the glossary) are, in essence, networks within nodes of networks (e.g., food webs within connected ponds; figure 1a). These networks and topologies can be combined as layers (a layer corresponds to one network) in multilevel or multilayer networks (see the glossary). For example, a network can include layers of different species interactions (e.g., antagonistic, mutualistic) that are linked to each other by species nodes (i.e., multiplex networks) and of human interactions, such as communication between managers (within-layer links; figure 1b) and how humans interact with the different species (between-layer links; figure 1b).

Social–ecological networks can therefore use different combinations of the concepts above but are, in essence, networks that integrate actors or entities (nodes) from both the social and ecological realms, interacting via social–social (SS), social–ecological (SE), and ecological–ecological (EE) links (*sensu* Bodin and Tengö 2012; see figure 1b for an example). Social–ecological networks have been used to better understand nature’s contributions to people (Dee et al. 2017, Felipe-Lucia et al. 2022), to improve sustainable resource use (Ortiz and Levins 2017, Zador et al. 2017, Barnes et al. 2019), and to inform measures for climate-change adaptation (Salgueiro-Otero et al. 2022).

We carried out a scoping literature review (for details, see supplement S2) and found 30 studies applying social–ecological networks to problems involving biological invasions. These studies applied a broad range of approaches to constructing and analyzing networks, stemming from different fields and theories. Eighteen studies (table 1) used networks with interactions between actors (nodes), including biophysical and social entities, on the basis of graph theory. The remaining 12 studies (see supplement S2, table 1) applied a range of tree graphs, causal influence diagrams (i.e., causal networks; see the glossary in supplement S1), semantic networks, and decision-making diagrams, as well as five studies using Bayesian networks (see the glossary). Given the vast range of possible approaches to social–ecological networks, in the following sections, we will focus on those that seem most promising for invasion science.

## Constructing and analyzing social–ecological networks in an invasion context

**Figure 2.**
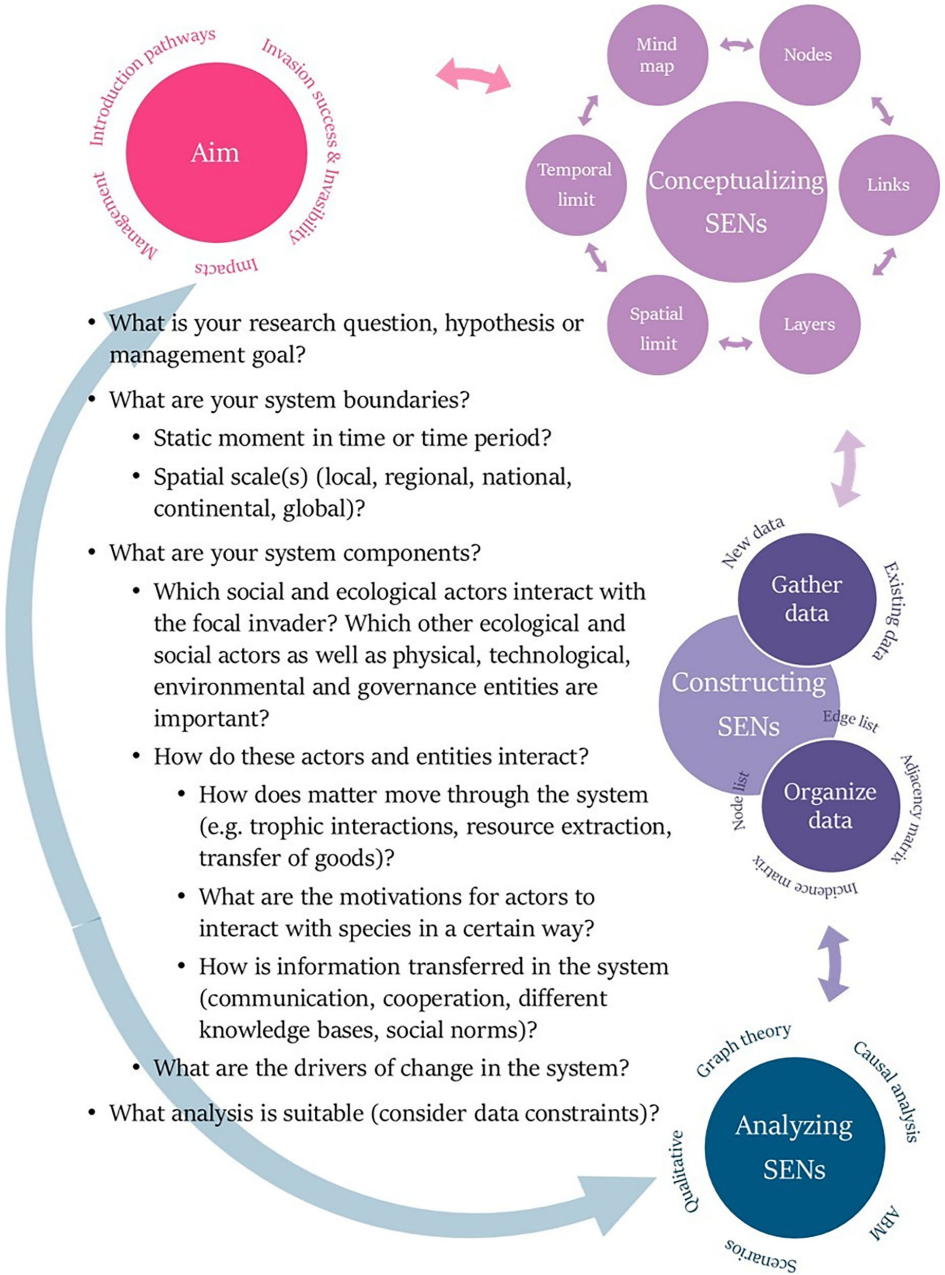
A flowchart depicting the iterative steps involved in conceptualizing, constructing, and analyzing social–ecological networks (SENs) (from the top to the bottom), as well as guiding questions (center).

The start of all social–ecological networks is a clearly defined aim—a research question, hypothesis, or management goal. Based on this aim, the social–ecological system under study should be conceptualized and characterized in an iterative process (figure 2).

### Step 1 : Conceptualizing social–ecological networks

When conceptualizing, prior knowledge or sufficient time to investigate the social–ecological system is needed to identify and define the system boundaries and components. Ideally, this knowledge is coproduced with stakeholders within the system (Moallemi et al. 2023). The temporal and spatial limits of the study should be specified prior to data collection. Depending on data availability, however, these limits may need to be adjusted throughout the study. The different actors or entities and interactions within the system must be defined in terms of nodes and links (figure 1b). If relationships are causal (such as impacts), a causal influence diagram can be constructed. Noncausal relationships and interactions, such as movement or communication, are frequently included in social–ecological networks as directed or undirected links (table 1). Alternatively, multilevel networks can help incorporate the many different interactions and actors, and for analyses based on graph theory, each layer within social–ecological networks corresponds to one type of link (figure 1b). Identifying and defining the relevant system components can be aided with guiding questions (figure 2) and linked back to the aim.

With all their layers and components, social–ecological networks offer several ways to include nonnative species (figure 1b): as nodes within the ecological network, as node attributes (see step 2 for details on attributes) of an invaded habitat node, or as links—for example, if the research aims to model the spread of a nonnative species across a landscape comprising discrete habitat patches serving as nodes. Nonnative species can also be modeled as link attributes of an infected vector, which, when made dynamic, models how nonnative species can move through the network of interactions (as in a contagion network). All the above-described components can be contemplated conceptually, but social–ecological networks should be simplified to an appropriate level of complexity, considering the aim and available resources.

### Step 2 : Constructing social–ecological networks

Following conceptualization, the underlying data for the nodes and links must be gathered. Existing data from databases, impact assessments, or grey and scientific literature can be used, as well as newly collected data. Interviews and surveys can provide valuable insights from stakeholders within the system under study. The data must then be organized in a network structure to allow for the subsequent analysis. Adjacency and incidence matrices (see the glossary in supplement S1) are sometimes used, but we will focus on node and edge lists in the present article (see the glossary in supplement S1). Node lists contain all node IDs as the first column (each row being one node), and the subsequent columns can contain different attributes of this node. Node attributes constitute any other relevant information or characteristic pertaining to the node—for example, demographics for social nodes, population densities for species, or other quantitative or qualitative variables. The corresponding edge list contains the pair of nodes corresponding to each link in the first two columns (each row represents one link), and the subsequent columns can contain link attributes (i.e., any other relevant information one wishes to include). For multilayer networks, each layer can be considered separately and characterized by its own node and edge list. Alternatively, the layer identity can be recorded as a node or edge attribute.

### Step 3 : Analyzing social–ecological networks

Social–ecological interaction networks can be analyzed topologically by identifying different attributes and structures within the network. Centrality measures (see the glossary in supplement S1), such as degree (the number of direct connections a node has) or closeness (how easily a node can reach all other nodes in the network on the basis of the shortest path), reflect a node’s relative importance in the network. Diameter, density, (average) path length and transitivity are topological network metrics (see the glossary) that can be used to understand and compare network attributes. These metrics can be linked to different theories and frameworks in the social and natural sciences (e.g., see above; Biggs et al. 2021, Hui and Richardson 2022, McLevey et al. 2024). Finding groups in networks can be done by applying algorithms such as walk trap, page rank, or random walk (cf. Farine and Whitehead 2015, Hashemi and Darabi 2022), and dominator tree analysis can identify bottlenecks within directed networks (e.g., Kluger et al. 2019). Motifs (see the glossary) are specific recurring patterns of interconnections (subgraphs), consisting of the specific configuration of links among two, three or more nodes (Milo et al. 2002). They can inform on actors’ abilities to manage shared resources (Bodin and Tengö 2012) and on social–ecological fit (Epstein et al. 2015, Guerrero et al. 2015, Bodin et al. 2016).

Motif analysis can be done by comparing the number of motifs in social–ecological networks with a random network or, for example, by using exponential random graph models where varying levels of randomness can be controlled for and where node attributes can be accounted for (see, e.g., McLevey et al. 2024). Such models can also be used to analyze how the network structure arose (using the network as the response variable), how the network structure contributes to certain phenomena (using the network as a predictor variable), or how links are likely to emerge given the existing structure (like a simulation). Other types of models used for network analysis include contagion or diffusion models, where the spread of something (e.g., money, influence, nonnative species) across a network is analyzed (e.g., Haak et al. 2017). Block modeling looks at the position of structures within multirelational (or multilevel or multilayer) networks (e.g., Harrer and Schmidt 2013), whereas agent-based models (e.g., Baggio et al. 2016) permit analysis of multiple interrelated processes and can either be used to explain how a network was formed or create network-based scenarios.

A breadth of theories and frameworks from invasion science, social–ecological systems research and other disciplines can be applied in combination with the social–ecological network approach (Biggs et al. 2021, Hui and Richardson 2022). In addition to insights already gained on how nonnative species affect food webs, the concept of social–ecological fit (see the glossary in supplement S1), stemming from social–ecological systems research, seems particularly useful. It refers to analyzing whether the ecological interdependencies are mirrored or complemented by the managing social structures (e.g., Alexander et al. 2017). For example, if connected invaded habitats are managed by two different social actors, it is key that these actors at least communicate, if not cooperate in order to match the ecological interdependencies. If no interaction between the social actors occurs, management efforts are unlikely to be effective, as reinvasions from the respective habitat patches may occur, or different management actions counteract each other.

All network types, both causal and interaction networks, can be analyzed using path analysis, which explores how to get from point A to B in a network and gives insight into connectivity and indirect effects. Loop analysis (i.e., a specific type of path analysis; see the glossary in supplement S1) is applicable to networks containing cycles, evaluating how one completes a loop from point A and back via other nodes and links in the network. Causal loop analysis can give insight into the stability of a system and the direct and indirect effects of external perturbations (stressors) (Levins 1974). More specifically, does a change in one state variable (node) increase, decrease, or have no effect on the other state variables in the system? The benefit of loop analysis is the relatively low resolution of data required (whether the effect of the interaction on the state variables is positive, negative, or neutral) and the ability to consider the system as a whole. In the context of biological invasions, causal loop analysis can be performed to understand whether nonnative species contribute to positive or negative feedback loops, what happens if interactions change (i.e., go from positive to negative or neutral and vice versa) and with which changes in state variables (nodes) and interactions (links) the system loses stability (Scotti et al. 2020).

A multitude of software packages from different disciplines exist to analyze networks. Food webs can, for example, be analyzed as mass-balanced models using Ecopath with Ecosim (Christensen and Pauly 1992, Christensen and Walters 2004) or EcoNet (Kazancı 2007); neural or genetic networks can be analyzed with software such as Cytoscape (https://cytoscape.org); and examples of software packages from the social sciences that help gather, organize, and analyze data are Gephi (https://gephi.org) and UCINET (Borgatti et al. 2002) with integrated NetDraw (Borgatti 2002). Alternatively, R provides many packages to visualize and analyze different network types—for example, enaR (Borett and Lau 2014 ), an ecosystem network analysis package, or igraph (Csárdi et al. 2025), which allows the assembly of a network item on the basis of node and edge lists, as well as adjacency and incidence matrices, and many tools to characterize, quantify, and visualize observed network structures and compute their metrics. The ggraph package (Pedersen 2017) offers additional visualization options, based on ggplot2 (Wickham 2016). Many more options for network analysis across multiple formats exist; see the curated list of Awesome Network Analysis (Briatte et al. 2024).

## An illustrative example: The impacts of nonnative species in Hawaii

Our aim is to map the cumulative direct and indirect impacts of nonnative vertebrate species in Hawaii and the underlying impact mechanisms, based on S/EICAT(+) assessments.

### Conceptualizing social–ecological networks of nonnative species impacts in Hawaii

Like many other oceanic islands, a high proportion of Hawaii’s native species are endemic. These species are mainly birds (98% are endemic), fishes, and invertebrates; Hawaii has no native terrestrial reptiles or amphibians and just three native terrestrial or semiterrestrial mammals (Pratt et al. 2009, MMC 2024). Of the 1456 native species that occur naturally in Hawaii, 32% are endangered, and many others have already gone extinct (US Fish and Wildlife Service 2024). Some Hawaiian people have a strong cultural connection with the archipelago’s wildlife and landscapes; this is reflected in Hawaiian art, music, traditional dress, and practices (Anderson-Fung and Maly 2002). For example, the feathers of native birds are used in rituals as headdresses and also as currency, and some native birds such as the ‘alalā (Hawaiian crow *Corvus hawaiiensis*) feature in stories and legends and as spiritual guides and protectors (Pratt et al. 2009). Indeed, the ‘alalā is considered to be a guardian of the soul on Hawaii; the souls of those that die on Ka‘ū (Hawaii Island) travel to a Leaping Place, which is a cliff, from where they are guided to their final resting place. Their guide is the ‘alalā and yet this species is now extinct in the wild (Rundell 2022).

The decline of endemic species in Hawaii has several anthropogenic causes, one of which is the introduction of nonnative species. For example, nonnative trees are replacing native canopy trees in all Hawaiian forests types (Potter et al. 2023), whereas invasive grasses inhibit native forest restoration (Rehm et al. 2023). Nonnative animals include several rat species (*Rattus* spp.) and the small Indian mongoose (*Urva auropunctata*), which prey on a range of native species, including the threatened Hawaiian stilt (*Himantopus mexicanus knudseni*); the barn owl (*Tyto alba*), which preys on native birds including the endangered Hawaiian petrel (*Pterodroma sandwichensis*); and the Japanese white-eye (*Zosterops japonicus*), which competes with native bird species, possibly threatening the Hawaii akepa (*Loxops coccineus*) with extinction, and which is believed to spread avian malaria, which affects many native bird species (Freed et al. 2008, Kaushik et al. 2018, Raine et al. 2019). The impacts of nonnative species have resulted in losses to ecosystem services provided by native species in Hawaii. For example, the islands have lost many native frugivorous birds that disperse the seeds of native plants—resulting in negative environmental impacts such as reduced habitat quality, (nonnative bird species have partially taken over this ecosystem function; however, they disperse a higher number of nonnative than native plant seeds; Vizentin-Bugoni et al. 2019) and cultural losses associated with the disappearance of these bird species. Many nonnative species are now established in Hawaii, including birds and mammals, but also terrestrial reptiles and amphibians. Their impacts on people and wildlife are widespread and diverse.

We identified impacts (links) associated with specific groups of native and nonnative species (nodes), creating a social–ecological network for impacts on native birds that are caused by nonnative vertebrate species, impacts affecting native species that are caused by nonnative birds, and the wider positive and negative socioeconomic impacts of these nonnative vertebrates on social entities (nodes) in Hawaii (spatial limit), from 1970 until present day (temporal limit). As the network of impacts is causal, layers do not need to be defined, although they are implied by the social and ecological nodes.

### Constructing social–ecological networks of nonnative species impacts in Hawaii

The biodiversity and socioeconomic impacts of nonnative species were identified by reviewing literature reported in the IUCN Global Invasive Species Database (www.iucngisd.org/gisd) and two global assessments of the environmental and socioeconomic impacts of nonnative birds (Evans et al. 2016, 2020) following the S/EICAT(+) frameworks (Blackburn et al. 2014, Bacher et al. 2018, IUCN 2020, Vimercati et al. 2022, https://doi.org/10.32942/X28M09 [preprint: not peer reviewed]). Notice, therefore, that both deleterious and beneficial environmental impacts are included in the analysis. An additional online search for cultural and socioeconomic impacts was carried out using Google and Google Scholar platforms from March to May 2024. The indirect impacts were explored by identifying the socioeconomic relevance of impacted native species.

The layers and nodes therefore consist of three types: native species, nonnative species, and social entities such as stakeholders and cultural practices. Native species were aggregated into forest and grassland birds, sea birds, and wetland birds, as well as plant communities. The nodes were assembled in a node list (supplement S3, table 1), with columns containing the layer or node type, the name of the node and the individual species within the groups. Nonaggregated nodes were given the value 1, and aggregated nodes the value of the respective number of species within that group. This was later used to scale the relative node sizes.

The links were the different beneficial and deleterious impacts and underlying mechanisms of nonnative species as well as the indirect impacts of these. An edge list (supplement S3, table 2) was created, containing the list of interactions (i.e., the node causing the impact, the node being impacted) and information describing it, including the type of impact, its mechanism, the number *n* of species impacted or causing the impact (i.e., the weight of the link), whether the impact and mechanisms were beneficial or deleterious to native species and valued aspects of Hawaiian culture, and whether the impact was actually observed (on the basis of evidence included in previous S/EICAT(+) assessments and published studies) or potential (on the basis of grey literature).

### Analyzing social–ecological networks of nonnative species impacts in Hawaii

**Figure 3.**
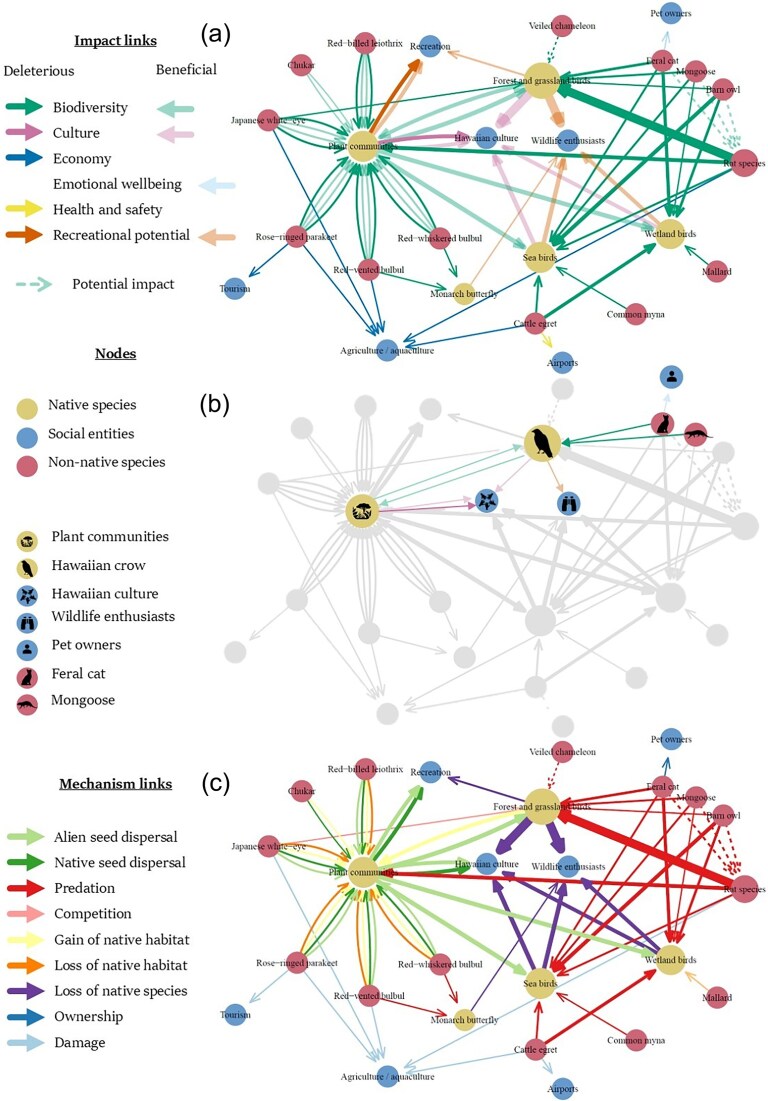
Case study illustrating the construction of social–ecological networks with a focus on impacts of nonnative vertebrates in Hawaii on native species and people, based on S/EICAT(+) assessments (see supplement S3 for details on the methodological approach). Nonnative vertebrates are indicated as red nodes: barn owl (*Tyto alba*), cattle egret (*Bubulcus ibis*), Japanese white-eye (*Zosterops japonicus*), red-billed leiothrix (*Leiothrix lutea*), red-vented bulbul (*Pycnonotus cafer*), red-whiskered bulbul (*Pycnonotus jocosus*), rose-ringed parakeet (*Psittacula krameri*), mallard (*Anas platyrynchos*), common myna (*Acridotheres tristis*), chukar (*Alectoris chukar*), feral cat (*Felis catus*), small Indian mongoose (*Urva auropunctata*), and the rat species brown rat (*Rattus norvegicus*), black rat (*Rattus rattus*), Polynesian rat (*Rattus exulans*); native species are indicated as yellow nodes: birds, selected invertebrates, plant communities, and people, including socioeconomics, are indicated as blue nodes. Network (a) depicts beneficial and deleterious social–ecological impacts (lighter shaded links indicate beneficial impacts), network (b) highlights in color the subset of impacts relating to the Hawaiian crow (“Alalā; *Corvus hawaiiensis*), and network (c) depicts impact mechanisms. In these networks, the thickness of the links (width of connecting arrows) indicates the number of native species affected. Several native species were aggregated into nodes (reflected by larger node size) representing taxonomic or functional groups; these nodes contain the following native species: Forest and grassland birds: akikiki (*Oreomystis bairdi*), Hawai’i ‘akepa (*Loxops coccineus*), Hawai’i ‘elepaio (*Chasiempis sandwichensis*), Hawaiian short-eared owl (pueo; *Asio flammeus sandwichensis*), palila (*Loxioides bailleui*), O’ahu ‘elepaio (*Chasiempis ibidis*), Hawai’i creeper (*Manucerthia mana*), ‘akohekohe (*Palmeria dolei*), kākāwahie (*Paroreomyza flammea*), O’ahu ‘alauahio (*Paroreomyza maculata*), Maui parrotbill (*Pseudonestor xanthophrys*), ʻōʻū (*Psittirostra psittacea*), Laysan finch (*Telespiza cantans*), Hawaiian crow (‘alalā; *Corvus hawaiiensis*); sea birds: brown noddy (*Anous stolidus*), Bulwer’s petrel (*Bulweria bulwerii*), Hawaiian petrel (‘ua‘u; *Pterodroma sandwichensis*), bonin petrel (*Pterodroma hypoleuca*), Newell’s shearwater (ʻaʻo; *Puffinus newelli*), wedge-tailed shearwater (*Ardenna pacifica*); wetland birds: Hawaiian common moorhen (‘alae ‘ula; *Gallinula chloropus sandvicensis*), Hawaiian coot (‘alae ke‘oke’o; *Fulica alai*), Hawaiian duck (koloa; *Anas wyvilliana*), Hawaiian goose (nēnē; *Branta sandvicensis*), Hawaiian stilt (ae’o; *Himantopus mexicanus knudseni*); plant communities: ‘ala ‘ala wai nui (*Peperomia subpetiolata*), Hawai’i cheesewood (*Pittosporum hawaiiense*), hō’awa (*Pittosporum napaliense*), pilo kea lau li’i (*Platydesma rostrata*), hala pepe (*Pleomele fernaldii*), opuhe (*Urera kaalae*).

This case study illustrates how existing data can be organized and visualized as relational data in a network; we focused in the present article on analyses for network visualization. Details on the R packages used for network visualization can be found in supplement S3. Three networks were plotted, with links showing either the impacts of nonnative species (figure 3a, 3b is focused on a subset of impacts relating to the Hawaiian crow) or the underlying mechanisms (figure 3c).

The ‘alalā (Hawaiian crow) is a native forest bird species that spreads the seeds of fruits—an important function for forest habitat maintenance and promoting biodiversity, as well as being culturally important and valued by bird watchers and wildlife enthusiasts (figure 3b). It is threatened by a range of nonnative species, including the small Indian mongoose (*Urva auropunctata*) and feral cats (*Felis catus*; figure 3b), because of predation (figure 3c)—particularly of fledglings which remain near the ground for several days before being able to fly—as well as habitat loss and other compounding factors. The disappearance of this culturally significant guardian of the soul on Hawaii can largely be attributed to the impacts of nonnative species, posing subsequent losses to biodiversity, culture, and recreation, affecting nature and people on Hawaii in different but connected ways. This is especially relevant as efforts to reestablish wild populations are ongoing (https://dlnr.hawaii.gov/alalaproject).

Mapping all recorded impacts (figure 3a) shows, for example, the cumulative indirect impacts of the loss of native bird species on Hawaiian culture that were caused, at least in part, by nonnative species (thicker lines indicate more impacts; figure 3a). Beneficial impacts were visually displayed to be more transparent than deleterious impacts, so that they could be distinguished by the reader. Figure 3a shows that the deleterious impacts of nonnative species on native species indirectly negatively impacting culture and recreation. Although S/EICAT(+) data do not consider (direct or indirect) impacts from one nonnative species on another, visualizing this data as a network shows exploitative and apparent competition going on between different nonnative species (figure 3c). Another aspect that becomes apparent when visualizing the data in this manner is that only impacts *on* social entities are considered. How social entities affect both native and nonnative species is not captured by S/EICAT(+) alone. If the causal links between social entities and ecological aspects of the system were to be mapped, loop analysis could be applied to identify positive and negative feedback loops (i.e., indirect impacts) that could aid or hinder management of nonnative species.

## Opportunities for social–ecological networks in invasion science

The social–ecological network methodology enables insights into varying aspects of invasion science. In principle, social–ecological networks can give insights into introduction pathways, invasion success, the invasibility of a system, impacts, and the management of nonnative species (i.e., the major themes of invasion science; cf. Musseau et al. 2024), as these aspects are intrinsically linked to one another. Which specific aspects social–ecological networks address is based on how nodes and links are defined, and what data are included. In the following, we present particularly promising opportunities of social–ecological networks in invasion science.

Past, present, and future introduction pathways of nonnative species can be modeled using spatial networks, with nodes representing spatially discrete regions and links indicating human-mediated dispersal (table 1). This has been done for trade networks to investigate pathways and therefore possible introduction risks, such as for the pet trade (Sinclair et al. 2021) or for pest species associated with the cassava trade (Wyckhuys et al. 2018). It has also been done to identify possible dispersal hubs of marine nonnative species on the basis of ship movements (Letschert et al. 2021) or secondary introductions through—for example, angler movement between lakes (the blue highlights in table 1). To better understand the conditions that lead to successful introductions, networks of the cultural and economic contexts of invasions can be constructed, such as by comparing nonnative flora similarities across former empires in the context of colonialism (Lenzner et al. 2022). Therefore, links between locations could account for similarities, transport routes or many layers of relations in a multiplex network. Alternatively, node attributes of locations can reflect conditions of introduction in the above-described networks. This can facilitate the identification of factors that determine successful introductions of nonnative species, ultimately serving as a suitable tool for risk assessment.

Location-specific social–ecological networks can inform on the invasion success of different species, as well as the invasibility of the invaded social–ecological system. Nested networks allow modeling a specific social–ecological network within each node of a spatially connected network (as was described above). Building on Haak et al. (2017), food webs nested within lakes and expanding to larger spatial scales, movement between locations could be combined with specific social–ecological networks for each location. For example, trophic interactions within a food web may give insight into the biotic resistance of a system, whereas additional interactions with humans (e.g., whether humans use the nonnative species, find it charismatic, or have any precautions against it) allow for a better understanding of the mechanisms surrounding invasion success and invasibility. Combining transport networks (which generate propagule pressure and therefore increase invasion success; cf. Jeschke and Starzer 2018) with information about local systems and their context along the invasion stages into one cohesive network will allow for more holistic insights into the invasion process and what determines successful invasions. It can also inform risk assessment and management.

Mapping out and visualizing the interactions and effects of invasive species shows the cumulative, direct, and indirect invader impacts and can be used as a communication tool to foster knowledge exchange, aid decision-making among stakeholders, and increase public awareness. Figure 3 depicts the social–ecological system surrounding nonnative species in Hawaii, highlighting the interconnectedness of different system components. Specifically, it highlights that one direct impact may have many indirect impacts and that when managing nonnative species, it is not sufficient to simply, for example, focus only on negative impacts caused by predation of a single nonnative species but to also consider management implications in the wider network. Achieving a shared understanding among stakeholders of the complexity and interdependence of the systems we inhabit is crucial for mitigating the impacts of nonnative species and other anthropogenic drivers of change.

Networks can be used to simulate different future scenarios and make predictions that can inform policy and management. The efficiency of invasive species management under different scenarios has been assessed using agent-based models (Yletyinen et al. 2021), and how people will react to new environmental conditions has been modeled using scenario-based adaptation pathways (Salgueiro-Otero et al. 2022). Many more possibilities for network-based scenarios exist, such as causal influence diagrams for analyzing the impact of different changing environmental factors on Alaskan forests (Wolken et al. 2011) and predicting the impacts of people’s perception on “nuisance” plant management using Bayesian belief networks (Thiemer et al. 2023). Interaction networks can be made dynamic with longitudinal data (i.e., different networks for different time points), thereby synthesizing historic development and supporting predictions of how the network may change in the future. This can also be done by specifically adding and removing nodes and links. For example, a nonnative species can be added as an additional node with its potential (i.e., biologically plausible) interactions (links) (cf. Penk et al. 2017, Fumero-Andreu et al. 2024) or impacted species can be removed to simulate extinctions and examine the resulting structural changes (i.e., network metrics). Alternatively, loop analysis can be used to simulate the knock-on changes within a network when interactions (links) and state variables (nodes) change.

Identifying the differences and similarities across different invaded social–ecological systems can give important insights into effective management along the invasion process. Network metrics enable the comparison of vastly different systems, assuming the network is similarly conceptualized and constructed using the same type of system components (nodes and links). This can be done to investigate why a nonnative species is or is not able to establish in different systems, what governance structures lead to better management, and why management of a nonnative species in one region is more effective than in another (Sandström and Rova 2010, Alexander et al. 2015, Alexander et al. 2017). Other comparative methods include weighted topological overlap, which directly compares the structure of two networks, or clustering coefficients such as modularity and density (see the glossary in supplement S1), which are just some of the network metrics that can give insight into how tightly connected a network is (Gysi and Nowick 2020). The frequency of specific motifs can also be compared across networks; however, theory on what these motifs mean in an invasion context must be developed. Building on biotic resistance and theory on environmental governance, we can assess which social–ecological network structures prevent or facilitate invasions, as well as which structures contribute to successful impact mitigation.

Social–ecological networks can also provide recommendations on how to positively transform invaded social–ecological systems. By inducing regenerative dynamics, disturbed systems can be transformed to reach a more desirable state. Regenerative dynamics are a type of positive feedback where a small change triggers a reinforcing cycle, leading to a spiral of improvement, as has been observed in some rewilding experiments (Fischer et al. 2024). These positive feedback loops, or virtuous cycles, can be identified using loop analysis (Scotti et al. 2020), as can the corresponding negative feedback loops, or vicious cycles. By recognizing important feedback loops in the system and running scenarios for improvement, we can identify leverage points for positive transformation.

Although social–ecological networks offer numerous benefits, the approach also presents several challenges and needs for further development in areas with relevance to invasion science. Specifically, social–ecological networks require a considerable amount of data; data extraction and subsequent analysis can be time consuming. On the other hand, the advantage of networks is that they can be continuously expanded. This makes them dynamic and can improve their accuracy over time and space. If the focal research question is sufficiently specific, social–ecological networks can be of tractable complexity, enabling more sophisticated analyses of, for instance, well-defined subsystems. The data hunger of social–ecological networks is becoming less problematic in the current age of big data. Large language models and other AI tools might provide additional support in this context, either by streamlining data collection from different sources or by inferring interactions, on the basis of traits and other relevant information (e.g., Fricke et al. 2022). It is also important to realize that social–ecological networks can serve as powerful synthesis tools for integrating different data and information sources and extracting key insights relevant to researchers across disciplines and diverse stakeholder groups, facilitating inter- and transdisciplinary exchange. Social–ecological networks explicitly facilitate the incorporation of different perspectives and can be used as tools for turning data and information into knowledge (cf. Jeschke et al. 2019).

## Conclusions

Given all the potential nodes and links that can be included in a network, from species to governing bodies, energy to causation, and a vast range of analysis methods already developed in different fields, the social–ecological network methodology allows for a more comprehensive understanding of biological invasions. Social–ecological networks that incorporate nonnative species can inform risk assessment, risk management, and model future scenarios. They can be used as a synthesis tool, as well as to communicate and engage with stakeholders to raise awareness and improve management. It is time to more fully explore the many opportunities of social–ecological network analyses for biological invasions, as these pose great potential in tackling the complex interactions and impacts of nonnative species. The ability of networks to, in principle, incorporate all relevant system components, throughout different spatial and temporal scales, enables a holistic analysis of social and ecological interdependencies within real-world invaded systems, subject to multiple drivers of change.

There is no single right way to construct and analyze social–ecological networks, but crucial decisions must be made on which system components to include and how to define network boundaries. Assumptions that one inevitably makes about the focal system should be based on prior knowledge of the system, ideally drawing on insights of actors that are part of the system, and by conducting participatory research. Social–ecological networks force us to explicitly consider the interactions within and across what were previously considered fundamentally different components of the human–nature relationships associated with nonnative species. By connecting different disciplines, engaging with diverse stakeholders, and synthesizing knowledge across realms, social–ecological networks will support our efforts to better understand biological invasions and their impacts, as well as how to improve their management.

## Supplementary Material

biaf174_Supplemental_Files

## Data Availability

The data underlying this article are available in the article and in its online supplementary material.

## Author Biography

Fiona S. Rickowski (fionarickowski@web.de), Florian Ruland, Rafael L. Macêdo, Robert Arlinghaus, Gustavo A. Castellanos-Galindo, James W. E. Dickey, Sabine Hilt, Yuval Itescu, Sophia Kimmig, Lohith Kumar, Florencia A. Yannelli, and Jonathan M. Jeschke are affiliated with the Leibniz Institute of Freshwater Ecology and Inland Fisheries, in Berlin, Germany. Fiona S. Rickowski, Florian Ruland, Thomas Evans, Rafael L. Macêdo, Gustavo A. Castellanos-Galindo, James W. E. Dickey, Yuval Itescu, Sophia Kimmig, Lohith Kumar, Florencia A. Yannelli, and Jonathan M. Jeschke are affiliated with the Institute of Biology at Freie Universität Berlin, in Berlin, Germany. Fiona S. Rickowski, Florian Ruland, James W. E. Dickey, and Jonathan M. Jeschke are affiliated with the Berlin–Brandenburg Institute of Advanced Biodiversity Research, in Berlin, Germany. Florian Ruland and Menja von Schmalensee are affiliated with the West Iceland Nature Research Centre, in Stykkishólmur, Iceland. Örjan Bodin is affiliated with the Stockholm Resilience Centre, at Stockholm University, in Stockholm, Sweden. Thomas Evans is affiliated with le laboratoire Ecologie, Systématique et Evolution at Université Paris-Saclay, in Paris, France. Mike S. Fowler is affiliated with the Department of Biosciences, in the Faculty of Science and Engineering, at Swansea University, in Swansea, Wales, in the United Kingdom. Lotta C. Kluger is affiliated with the Center for Ocean and Society at Kiel University, in Kiel, Germany. Lotta C. Kluger is affiliated with the Department of Agricultural Economics at Kiel University, in Kiel, Germany. Guillaume Latombe is affiliated with the Institute of Ecology and Evolution at the University of Edinburgh, in Edinburgh, Scotland, in the United Kingdom. Guillaume Latombe, Bernd Lenzner, and Franz Essl are affiliated with the Division of BioInvasions, Global Change, and Macroecology, in the Department of Botany and Biodiversity Research at the University of Vienna, in Vienna, Austria. Tim Adriaens is affiliated with the Research Institute for Nature and Forest, in Brussels, Belgium. Robert Arlinghaus is affiliated with the Division of Integrative Fisheries Management, in the Faculty of Life Sciences at Humboldt-Universität zu Berlin, in Berlin, Germany. Gustavo A. Castellanos-Galindo is affiliated with the Smithsonian Tropical Research Institute, in Balboa, Panama. Jaimie T. A. Dick is affiliated with the Institute for Global Food Security, in the School of Biological Sciences at Queen’s University Belfast, in Belfast, Northern Ireland, in the United Kingdom. James W. E. Dickey is affiliated with the GEOMAR Helmholtz Centre for Ocean Research Kiel, in Kiel, Germany. and with the National Laboratory for Health Security, HUN-REN Centre for Ecological Research, in Budapest, Hungary. Belinda Gallardois affiliated with the Instituto Pirenaico de Ecología, at the Spanish National Research Council, in Zaragoza, Spain. Yuval Itescu is affiliated with the Department of Evolutionary and Environmental Biology at the University of Haifa, in Haifa, Israel. Ivan Jarić is affiliated with the Université Paris-Saclay, CNRS, AgroParisTech, Ecologie Systématique Evolution, in Gif-sur-Yvette, France. Ivan Jarić is affiliated with the Biology Centre of the Czech Academy of Sciences, at the Institute of Hydrobiology, in České Budějovice, in the Czech Republic. Lohith Kumar is affiliated with the ICAR-Central Inland Fisheries Research Institute, in Barrackpore, India. Ana Novoa, Petr Pyšek, and David M. Richardson are affiliated with the Department of Invasion Ecology, in the Institute of Botany at the Czech Academy of Sciences, in Průhonice, in the Czech Republic. Ana Novoa is affiliated with the Estación Experimental de Zonas Áridas, in the Consejo Superior de Investigaciones Científicas, in Almería, Spain and the Department of Invasion Ecology, in the Institute of Botany at the Czech Academy of Sciences, in Pruhonice, in the Czech Republic. Francisco J. Oficialdegui is affiliated with the Department of Conservation Biology and Global Change, Doñana Biological Station (CSIC), Seville, Spain. Francisco J. Oficialdegui is affiliated with Faculty of Fisheries and Protection of Waters, South Bohemian Research Centre of Aquaculture and Biodiversity of Hydrocenoses, University of South Bohemia in České Budějovice, České Budějovice, Czech Republic. Cristian Pérez-Granados is affiliated with the Biodiversity Conservation and Management Programme, at the Forest Science and Technology Center of Catalonia (CTFC), in Solsona, Catalonia, Spain. Petr Pyšek is affiliated with the Institute of Botany of the Czech Academy of Sciences, in Průhonice, andDepartment of Ecology, in the Faculty of Science at Charles University, in Prague, in the Czech Republic. Wolfgang Rabitsch is affiliated with the Environment Agency Austria, in Vienna, Austria. David M. Richardson is affiliated with the Centre for Invasion Biology, in the Department of Botany and Zoology at Stellenbosch University, in Stellenbosch, South Africa. Núria Roura-Pascual is affiliated with the Departament de Ciències Ambientals, in the Facultat de Ciències at the Universitat de Girona, in Girona, Catalonia, Spain. Florencia A. Yannelli is affiliated with the Argentine Institute for Dryland Research, at CONICET and the Universidad Nacional de Cuyo, in Mendoza, Argentina. Montserrat Vilà is affiliated with the Estación Biológica de Doñana, and with the Department of Plant Biology and Ecology at the Universidad de Sevilla, in Sevilla, Spain. Giovanni Vimercati is affiliated with the Department of Biology at the University of Fribourg, in Fribourg, Switzerland
