# Supplementary material for: A Systems Perspective: How Social–Ecological Networks Can Improve Our Understanding and Management of Biological Invasions
Source: Bioscience. 2025 Dec 4;76(2):127–46. doi: 10.1093/biosci/biaf174 (PMC12856202; doi:10.1093/biosci/biaf174)
Supplement: biaf174_Supplemental_Files [file biaf174_supplemental_files.zip › Supplement 1 - Glossary_r1.docx]

**Supplement 1: Glossary**

**Glossary containing key terms to the manuscript “A systems perspective: How social-ecological networks can improve our understanding and management of biological invasions” by Fiona Rickowski et al.**

**Adjacency matrix** – a data structure for the construction and analysis of networks. The first row and column is a repetition of all nodes, and the spaces in-between can have binary, categorical or continues variables representing the links.

**Agent-based models** – a computational model where actors (agents) interact with other actors and the environment (patches) according to sets of rules.

**Bayesian network** – a specific type of network using Bayesian statistics to model probabilities as links between variables (nodes).

**Bipartite network** – network with two types of nodes and where links only exist between these different node types.

**Causal network** – a network depicting causalities (links) between variables (nodes).

**Directed network** – a network where links can be uni- or bidirectional.

**Dynamic network** – a network changing through time.

**Equilibrium** – a theoretical state to which the system strives.

**Graph theory** – the study of structures within a network of interactions), based on Euler (1741)’s mathematical solution to wanting to find a path over 7 the bridges within the then town of Königsberg, that leads across every bridge only once.

**Incidence matrix** - a data structure for the construction and analysis of bipartite networks or between-layer links. The first row and column are different node types, and the spaces in-between can have binary, categorical or continues variables as the links.

**Layer** – a sub-network of a multilayer or multilevel network consisting of one node and link type, linked to other layers in the network.

**Link, edge or tie** – the interaction or relationship between two nodes within a network.

**Loop analysis** – a type of path analysis that examines how to get from one node in a network via other nodes back again. The network must be directed and have information on whether the effects of the interactions (links) between two state variables (nodes) is positive or negative, so if it increases or decreases the other state variables.

**Motifs** – small building blocks within networks consisting of the links between three or more nodes (if node attributes are accounted for, or if directed links are used, the number of nodes could be less than two).

**Multilayer network** - a type of network consisting of multiple sub-networks (layers) that contain the same set or subset of nodes of the same type, but where each layer consists of a specific type of link. A specific subset of multilayer networks, which contain all nodes in all layers as opposed to only subsets of these, are called multiplex networks.

**Multilevel network** – a type of network consisting of multiple sub-networks (levels or layers) that are connected to each other (between-layer links), where each layer consists of a specific type of node and link, and the links between the layers are also of a specific type.

**Multipartite network** – network with multiple types of nodes.

**Nested network** – network conceptually embedded within the nodes of other networks.

**Network metrics** – descriptive variables of networks, such as:

- **Centrality measures** – indicate the potential importance of different nodes based on their location in the network, including degree centrality, closeness centrality and betweenness centrality which indicate the potential importance of different nodes based on their location in the network.
- **Density** – proportion of links compared to the maximum possible number of links.
- **Modularity** – number of groups of nodes in the network, based on the density of links between nodes.
- **Path length** is the number of steps from one node to the other along existing links.
- **Diameter** – the shortest path length of all the longest possible paths without repetition through the network.
- **Transitivity** – a measure of connectivity relating to the probability of adjacent nodes being connected.

**Node or link attribute** – characteristics of the entities or relationships included in the node and edge list respectively, such as demographic variables or contamination of vectors.

**Node or vertex** – an actor or entity within a network.

**Social-ecological fit** – a theory from environmental governance research on how ecological connectivity (ecological links) should be mirrored or matched by the cooperation (social links) between governing bodies (social nodes) that manage (social-ecological links) ecological entities (ecological node i.e. habitat patch), in order to sustainably manage social-ecological systems.

**Social-ecological networks (SENs)** - models of social (human) and ecological (nature) interactions, consisting of nodes and links.

**Stability** – a property of a system where the system continues to function despite external stressors (i.e. change) affecting it.

**Unipartite network** – network with one type of node.

**Weighted network** – a network where the strength/magnitude of the links is quantified.
