# Supplementary material for: A Systems Perspective: How Social–Ecological Networks Can Improve Our Understanding and Management of Biological Invasions
Source: Bioscience. 2025 Dec 4;76(2):127–46. doi: 10.1093/biosci/biaf174 (PMC12856202; doi:10.1093/biosci/biaf174)
Supplement: biaf174_Supplemental_Files [file biaf174_supplemental_files.zip › Supplement 2 - Literature search_r1.docx]

**Supplement 2: Scoping literature review**

**Methods, results and additional examples of papers utilizing causal social-ecological networks to study biological invasions by Fiona Rickowski et al.**

A search in the Web of Science was conducted on 8 April 2024 with the following string, based on search strings applied by Evans et al. (2016) and Kluger et al. (2020):

(ALL=(“introduced species” OR “invasive species” OR “invasive alien species” OR “IAS” OR “alien” OR “non-native” OR “non-indigenous” OR “invasive” OR “pest” OR “feral” OR “exotic”)) AND

(ALL=(“social-ecological network” OR “socio-ecological network” OR “eco-social network”) OR ALL=(“ecological network” AND “social”) OR ALL=(“social network” AND “ecological”) OR ALL=(“social-ecological system” AND (“network approach” OR “network analysis” OR “network model”)) OR ALL=(“socio-ecological system” AND (“network approach” OR “network analysis” OR “network model”)) OR ALL=(“social-ecological” AND (“network approach” OR “network analysis” OR “network model”)) OR ALL=(“socio-ecological” AND (“network approach” OR “network analysis” OR “network model”)) OR ALL=(“eco-social” AND (“network approach” OR “network analysis” OR “network model”)) OR ALL=(“eco-social system” AND (“network approach” OR “network analysis” OR “network model)))

This search yielded 263 results which included two duplicates that were removed. The abstracts and titles of the remaining 261 publications were screened, and obvious mismatches were removed (e.g. papers on social interactions of ant colonies or router networks). The remaining 172 papers were checked extensively for the following criteria:

1. Non-native species explicitly as system component(s); AND
2. Relational data (qualitative or quantitative), either (a) explicitly defined as nodes, vertices or actors connected by links, edges or ties; or (b) visualized as a network; or (c) analyzed as a network (graph theory, etc.); AND
3. Both social and ecological system components, where social components can be humans or human-created entities and concepts, for example infrastructures, institutions, organizations or regulations; and ecological entities can be biophysical actors, entities or natural processes, for example non-human species, habitats or nutrient cycling.

This resulted in 22 studies, plus eight additional studies from an unsystematic search through google scholar, references within other papers and recommendations from colleagues over the period 2022-2024. These eight studies contained relevant examples, however due to keywords and the limitations of the Web of Science, they did not appear in the systematic search. Of these altogether 30 studies, 18 include graph theory based social-ecological networks examining interactions between actors. The remaining 12 studies examine causal effects and semantics, use Bayesian networks or classification trees and utilize visual benefits of networks (Supplementary 1, Table 1). While networks can be anything from a mind map, an ontology, a sociogram or the graphical depiction of causal relationships within a complex system, different disciplines use different terms and have developed different analysis techniques. Causal networks depict relationships between concepts and can be referred to as causal inference diagrams (CID), causal graphs or conceptual influence diagrams. Directed acyclic graphs (DAGs) are specific types of networks (or graphs) that have start- and endpoints as well as a direction and are frequently used for analyzing causality (Laubach et al. 2021).

Of the 18 studies applying a SEN analysis (Table 1, main manuscript), 13 focused on different aspects of invasive species whilst the remaining five studies included non-native species as secondary components related to other issues, such as alignment with institutional frameworks (two studies), management beyond invasive species control (two studies) and non-human agency (one study). The level of articulation i.e. how explicitly the social and ecological components are defined, sensu Kluger et al. 2020, ranged from one study including both ecological and social nodes as well as all links within and between these (articulation type III); to 10 studies including some, but not all social and ecological components and links (articulation type II); and seven studies considering social networks within an ecological context (six studies) or an ecological network based on stakeholders’ knowledge (one study; both articulation type I). Although the types of networks constructed, the different nodes and links defined, and the analyses performed vary greatly (Table 1), the two most common applications of SENs to invasion science to date are: (1) the human-aided spread of invasive species across a geographic region (nine studies), where nodes are specific locations and links the vectors of spread; and (2) the investigation of governance networks surrounding invasive species (six studies).


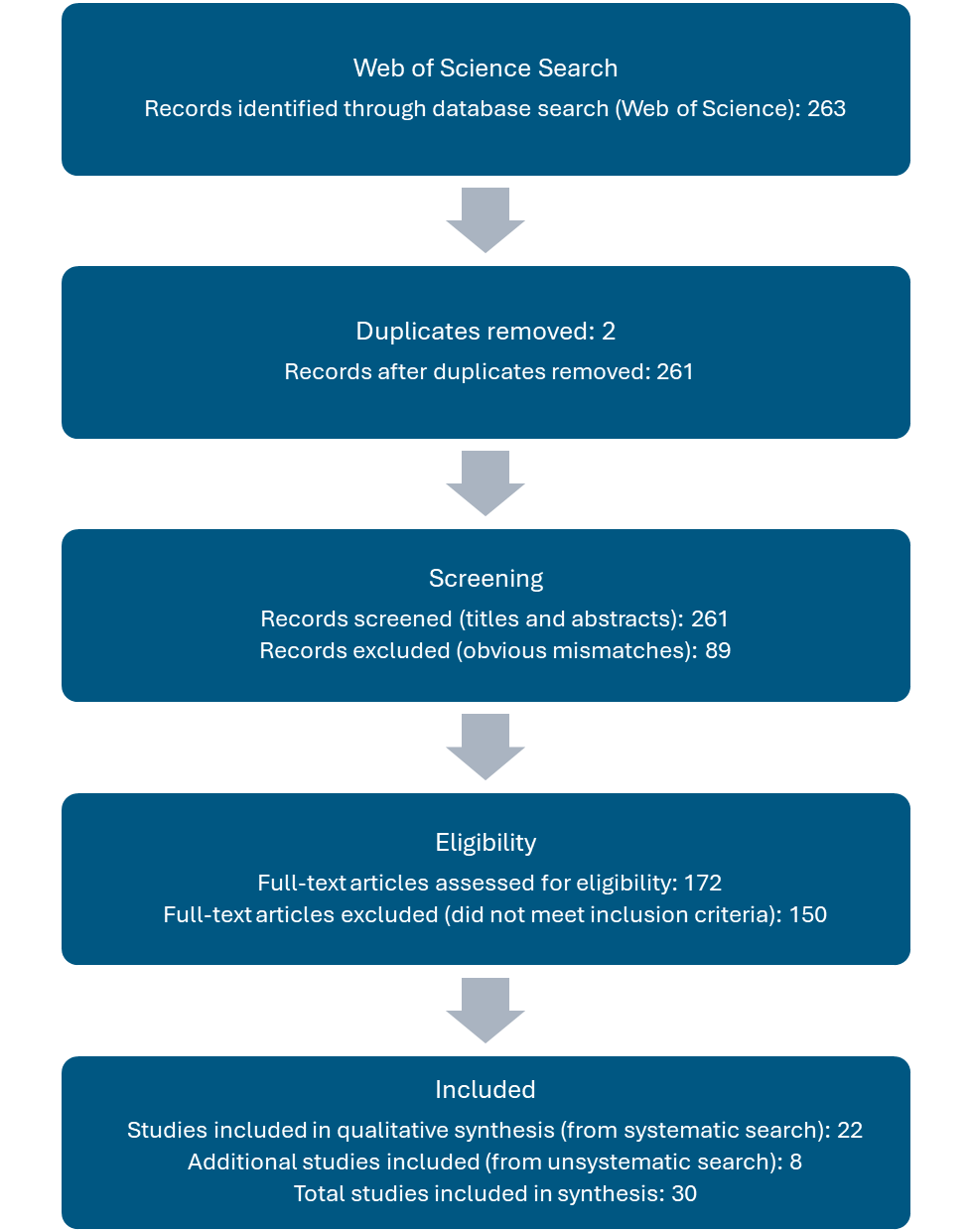


*Supplementary Figure 1: PRISMA flow diagram of scoping literature search for papers using social-ecological networks to study biological invasions.*

*Supplementary Table 1: Publications using other network approaches than social-ecological networks to study social-ecological relations involving invasive species (publications using social-ecological networks are included in Table 1 of the main article). Relevant selected themes in invasion science are based on Musseau et al. (2024; pathways; invasion success, incl. spread, and invasibility; impact; or management).*

| **Study** (^s^ and ^u^ indicate if found with systematic or unsystematic search) | **Research focus** | **Relevant theme(s) in invasion science** | **Network type** | **Nodes and links** | **Invasive species** | **Data source(s)** | **Analysis** | **Key findings in brief** |
| --- | --- | --- | --- | --- | --- | --- | --- | --- |
| Cidrás & González-Hidalgo 2022^u^ | Management of invasive species through sociocultural and stakeholder perspectives | Management | Tree graph | Nodes: stakeholders’ concepts of IAS  Links: relations to category | *Eucalyptus globulus*  Conceptual representations of invasive species as nodes in stakeholder networks | Survey; semi-structured interviews | Qualitative content analysis of survey and interview data | Activists in Galicia define *E. globulus* as invasive based on its non-native origin, rapid growth, poor forestry management, and its perceived cultural and landscape impacts |
| Drake et al. 2015^s^ | Introductions of invasive species | Invasibility, management | Risky behavior classification tree | Nodes: risky behaviors    Links: decisions | *Neogobius melanostomus*, *Bythotrephes longimanus*, and the viral hemorrhagic  septicemia (VHS) virus  Risky behaviors as nodes | Survey | Predictive models | Human behavior plays a crucial role in invasive species management, with prevention efforts hindered by persistent risky actions driven by misperceptions and external factors |
| Gonzalez et al. 2008^s^ | Adaptive co-management, social-ecological systems | Pathways, invasibility | (Causal Influence Diagram), signed directed graphs | Nodes: source, producer, consumer, tank  Links: influence | Unspecified; Invasive alien plants, insects, and native/endemic species  as social-ecological components | Participatory workshop, resilience theory application | Causal systems, adaptive cycles, plausible scenarios | Resilience-building through integrative management, tourism as a key driver |
| Lebel et al. 2010^s^ | Sustainable transition in shrimp aquaculture | Pathways, impact, management | Conceptual social-ecological network | Nodes: key events, farmers, policy  Links: policy-environment interactions, impacts of species replacements | *Litopenaeus vannamei*  as focus species as nodes in the transition framework | Databases, environmental indicators, interviews, grey literature (e.g., newspapers) | Qualitative analysis | Shift from black tiger to Pacific white shrimp improved resource efficiency but marginalized small producers, driven by disease management, global competitiveness, and certification |
| Luoma et al. 2021^s^ | Biofouling management, | Management | Causal influence diagram | Nodes: decision, chance, utility  Links: conditional dependencies (effects) | Unspecified, fouling species  as nodes | Scientific and grey literature, interviews | Qualitative; Bayesian Networks or optimization models only suggested for future studies | Trade-offs between hull coatings, in-water cleaning (IWC), and risks like NIS introduction and ecotoxicity. |
| Wolken et al. 2011^s^ | Climate change, , focusing on biophysical and social subsystem interactions | Management | Conceptual social-ecological interactions | Nodes: social- ecological system components  Links: interactions between system components | *Dendroctonus rufipennis*, *Monsoma pulveratum*, *Eriocampa ovata*, *Alliaria petiolata*, *Caragana arborescens*, *Crepis tectorum*, *Fallopia* spp., *Hieracium aurantiacum*, *Melilotus alba*, *Prunus padus*  as nodes | Literature, global climate  models | Qualitative, conceptual framework | Increased wildfires, insect outbreaks, invasive species, and altered hydrology can cause region-specific impacts, with cascading ecological and societal consequences. |
| Yletyinen et al. 2021^u^ | Management, stakeholder perceptions | Management | Decision-making diagram | Nodes: decision making, invasion dynamics, behavioral responses  Links: influence | *Pinus nigra, P. contorta*  as attribute in agent-based model | Survey | SEPIM (agent-based model), various management scenarios | Social and ecological processes interact dynamically, influencing control efficiency; early detection critical for success. |
| Bayliss et al. 2018^s^ | Climate change adaptation and invasive species management | Management | Bayesian belief network | Nodes: social-ecological system components  Links: positive and negative effects | *Sus*  *scrofa*, *Urochloa mutica*  as nodes | Scientific and grey literature, risk assessments | Bayesian belief network, management scenarios | Feral pigs and para grass threaten ecosystems, requiring adaptive, long-term management |
| Dutra et al. 2018^s^ | Climate change adaptation and invasive species management | Management | Bayesian belief network | Nodes: social-ecological system components  Links: positive and negative effects | Unspecified; feral and aquatic invasive species  as nodes | Existing diagnostic frameworks, monitoring data, and participatory workshops | Bayesian belief network; management scenarios | Adaptive strategies combining soft barriers, participatory monitoring, and governance improve SES resilience under saltwater intrusion |
| Langmead et al. 2009^s^ | Ecosystem management, eutrophication, social-ecological resilience | Management, | Bayesian belief network | Nodes: Socio-economic drivers, ecosystem components (abiotic and biotic)  Links: effects | Unspecified planktonic and benthonic invasive species  as nodes | Historical data, expert opinion, empirical time-series | Bayesian belief network; management scenarios | Socio-economic choices directly affect eutrophication, resilience, and recovery; adaptive policy integration is essential. |
| Salliou et al. 2017^s^ | Ambiguity and stakeholder perspectives in social-ecological systems | Management | Bayesian belief network | Nodes: stakeholder beliefs (conceptual node), landscape complexity, pests, predators, apple production  Links: interactions | *Cydia pomonella*, other pest species  as nodes | Expert elicitation, | Bayesian belief network, participatory modeling | Stakeholders' beliefs about landscape effects on pests and ecosystem services vary, highlighting the need for participatory approaches to resolve ambiguities. |
| Thiemer et al. 2023^u^ | Stakeholder perception of macrophyte growth and its implications for management | Management | Bayesian belief network | Nodes: macrophyte species, growth levels, respondent types, recreation activities  Links: conditional probabilities | *Egeria nuttallii*  *Sagittaria sagittifolia*  *Ludwigia* spp.  *Pontederia crassipes* (formerly *Eichhornia crassipes*)  *Juncus bulbosus*  as nodes | Surveys | Bayesian modeling (decision support tool) | Perceived nuisance varies by respondent type, activity, and macrophyte species; management strategies should account for local user preferences and ecological consequences. |

References

Bayliss P, Finlayson CM, Innes J, Norman-López A, Bartolo R, Harford A, Pettit NE, Humphrey CL, Van Dam R, Dutra LXC, Woodward E, Ligtermoet E, Steven A, Chariton A, Williams DK. 2018. An integrated risk-assessment framework for multiple threats to floodplain values in the Kakadu Region, Australia, under a changing climate. Marine and Freshwater Research 69: 1159–1185.

Cidrás D, González-Hidalgo M. 2022. Defining invasive alien species from the roots up: Lessons from the ‘De-eucalyptising Brigades’ in Galicia, Spain. Political Geography 99: 102746.

Drake DAR, Mercader R, Dobson T, Mandrak NE. 2015. Can we predict risky human behaviour involving invasive species? A case study of the release of fishes to the wild. Biological Invasions 17: 309–326.

Dutra LXC, Bayliss P, McGregor S, Christophersen P, Scheepers K, Woodward E, Ligtermoet E, Melo LFC. 2018. Understanding climate-change adaptation on Kakadu National Park, using a combined diagnostic and modelling framework: A case study at Yellow Water wetland. Marine and Freshwater Research 69: 1146–1158.

González JA, Montes C, Rodriguez J, Tapia W. 2008. Rethinking the Galapagos Islands as a complex social-ecological system: Implications for conservation and management. Ecology and Society 13.

Langmead O, McQuatters-Gollop A, Mee LD, Friedrich J, Gilbert AJ, Gomoiu MT, Jackson EL, Knudsen S, Minicheva G, Todorova V. 2009. Recovery or decline of the northwestern Black Sea: A societal choice revealed by socio-ecological modelling. Ecological Modelling 220: 2927–2939.

Laubach ZM, Murray EJ, Hoke KL, Safran RJ, Perng W. 2021. A biologist’s guide to model selection and causal inference. Proceedings of the Royal Society B: Biological Sciences 288: 20202815.

Lebel L, Mungkung R, Gheewala SH, Lebel P. 2010. Innovation cycles, niches and sustainability in the shrimp aquaculture industry in Thailand. Environmental Science and Policy 13: 291–302.

Luoma E, Nevalainen L, Altarriba E, Helle I, Lehikoinen A. 2021. Developing a conceptual influence diagram for socio-eco-technical systems analysis of biofouling management in shipping – A Baltic Sea case study. Marine Pollution Bulletin 170: 112614.

Salliou N, Barnaud C, Vialatte A, Monteil C. 2017. A participatory Bayesian Belief Network approach to explore ambiguity among stakeholders about socio-ecological systems. Environmental Modelling and Software 96: 199–209.

Thiemer K, Immerzeel B, Schneider S, Sebola K, Coetzee J, Baldo M, Thiebaut G, Hilt S, Köhler J, Harpenslager SF, Vermaat JE. 2023. Drivers of Perceived Nuisance Growth by Aquatic Plants. Environmental Management 71: 1024–1036.

Wolken JM, Hollingsworth TN, Rupp TS, Chapin FS, Trainor SF, Barrett TM, Sullivan PF, Mcguire AD, Euskirchen ES, Hennon PE, Beever EA, Conn JS, Crone LK, D’Amore DV, Fresco N, Hanley TA, Kielland K, Kruse JJ, Patterson T, Schuur EAG, Verbyla DL, Yarie J. 2011. Evidence and implications of recent and projected climate change in Alaska’s forest ecosystems. Ecosphere 2.

Yletyinen J, Perry GLW, Burge OR, Mason NWH, Stahlmann‐Brown P. 2021. Invasion landscapes as social‐ecological systems: Role of social factors in invasive plant species control. People and Nature 3: 795–810.
