# Supplementary material for: A Systems Perspective: How Social–Ecological Networks Can Improve Our Understanding and Management of Biological Invasions
Source: Bioscience. 2025 Dec 4;76(2):127–46. doi: 10.1093/biosci/biaf174 (PMC12856202; doi:10.1093/biosci/biaf174)
Supplement: biaf174_Supplemental_Files [file biaf174_supplemental_files.zip › Supplement 3 - Illustrative example_r1.docx]

**Supplement 3: Illustrative example**

**A social-ecological network of Hawaii based on S/EICAT(+) assessments and other literature sources, by Fiona Rickowski et al.**

The aim of this network was to effectively visualize and communicate (a) different cumulative direct and indirect impacts of non-native species (NNS) in Hawaii and (b) the underlying impact mechanisms. To not visually overcomplicate the networks, we chose to use node type to indicate the layers. The network visualizations were done with R version 4.3.2 (2023-10-31 ucrt) -- "Eye Holes". The R package *igraph* (Csardi & Nepusz 2006) was used to turn the edge and node list into a network item. The function *TKplot()* within *igraph* was used to manually lay out the network. The layout is an aesthetic attempt to visualize the networks in a straightforward manner that is easy to interpret, without being based on an algorithm or framework. The *TKplot* layout was then used in a *ggraph()* plot using the packages *ggplot2* (Wickham 2016) and *ggraph* (Pedersen 2022).

This example should serve as a proof of concept about the potential of data collected for S/EICAT(+) and other NNS impact assessments to create SENs, which can be useful to identify indirect impacts of NNS that typical impact assessments can miss. Due to the proof-of-concept nature of this example, we acknowledge that the data we collected does not include all interactions within the Hawaiian social-ecological system. For example, there are likely feedbacks from wetland birds and seabirds to plant communities, for instance through the nutrient loads of seabird guano being released into the ecosystem.

*Table 1: Complete node list for the Hawaiian S/EICAT(+) SENs shown in Figure 3 of the main text, with the different types of nodes (layers), the node names (node), the species contained within the nodes (species), and the respective sum (size).*

| **Layer** | **Node** | **Species** | **Taxa** | **Size** |
| --- | --- | --- | --- | --- |
| Non-native spp. | Barn owl | Barn owl (*Tyto alba*) | Aves | 1 |
| Non-native spp. | Cattle egret | Cattle egret (*Bubulcus ibis*) | Aves | 1 |
| Non-native spp. | Japanese white-eye | Japanese white-eye (*Zosterops japonicus*) | Aves | 1 |
| Non-native spp. | Red-billed leiothrix | Red-billed leiothrix (*Leiothrix lutea*) | Aves | 1 |
| Non-native spp. | Red-vented bulbul | Red-vented bulbul (*Pycnonotus cafer*) | Aves | 1 |
| Non-native spp. | Red-whiskered bulbul | Red-whiskered bulbul (*Pycnonotus jocosus*) | Aves | 1 |
| Non-native spp. | Rose-ringed parakeet | Rose-ringed parakeet (*Psittacula krameri*) | Aves | 1 |
| Non-native spp. | Mallard | Mallard (*Anas platyrynchos*) | Aves | 1 |
| Non-native spp. | Common myna | Common myna (*Acridotheres tristis*) | Aves | 1 |
| Non-native spp. | Chukar | Chukar (*Alectoris chukar*) | Aves | 1 |
| Non-native spp. | Feral cat | Feral cat (*Felis catus*) | Mammalia | 1 |
| Non-native spp. | Mongoose | Small Indian mongoose (*Urva auropunctata*) | Mammalia | 1 |
| Non-native spp. | Rat species | Brown rat (*Rattus norvegicus*)  Black rat (*Rattus rattus*)  Polynesian rat (*Rattus exulans*) | Mammalia | 3 |
| Non-native spp. | Veiled chameleon | Veiled chameleon (*Chamaeleo calyptratus*) | Reptilia | 1 |
| Native spp. | Forest and grassland birds | Akikiki (*Oreomystis bairdi*) Hawaii akepa (*Loxops coccineus*) Hawaii elepaio (*Chasiempis sandwichensis*) Hawaiian short-eared owl (Pueo) (*Asio flammeus sandwichensis*) Palila (*Loxioides bailleui*) Oahu Elepaio (*Chasiempis ibidis*) Hawaii creeper (*Manucerthia mana*)  Akohekohe (*Palmeria dolei*) Kakawahie (*Paroreomyza flammea*) Oahu Alauahio (*Paroreomyza maculata*) Maui parrotbill (*Pseudonestor xanthophrys*) Ou (*Psittirostra psittacea*)  Laysan finch (*Telespiza cantans*)  Hawaiian crow ('Alalā) (*Corvus hawaiiensis*) | Aves | 14 |
| Native spp. | Monarch butterfly | Monarch butterfly (*Danaus Plexippus*) | Invertebrata | 1 |
| Native spp. | Plant communities | ‘Ala ‘ala wai nui (*Peperomia subpetiolata*) Hawai'i cheesewood (*Pittosporum hawaiiense*)  Hō‘awa (*Pittosporum napaliense*) Pilo kea lau li'I (*Platydesma rostrata*) Hala pepe (*Pleomele fernaldii*) Opuhe (*Urera kaalae*) | Plantae | 6 |
| Native spp. | Sea birds | Brown noddy (*Anous stolidus*) Bulwer’s petrel (*Bulweria bulwerii*) Hawaiian petrel ('Ua'u) (*Pterodroma sandwichensis*) Bonin petrel (*Pterodroma hypoleuca*) Newell’s shearwater (ʻAʻo) (*Puffinus newelli*) Wedge-tailed shearwater (*Ardenna pacifica*) | Aves | 6 |
| Native spp. | Wetland birds | Hawaiian common moorhen ('Alae 'ula) (*Gallinula chloropus sandvicensis*) Hawaiian coot ('Alae ke'oke'o) (*Fulica alai*) Hawaiian duck (Koloa) (*Anas wyvilliana*) Hawaiian goose (Nēnē) (*Branta sandvicensis*) Hawaiian stilt (Ae'o) (*Himantopus mexicanus knudseni*) | Aves | 5 |
| Social | Agriculture / aquaculture | Human | Mammalia | 1 |
| Social | Airports | Human | Mammalia | 1 |
| Social | Wildlife enthusiasts | Human | Mammalia | 1 |
| Social | Hawaiian culture | Human | Mammalia | 1 |
| Social | Pet owners | Human | Mammalia | 1 |
| Social | Recreation | Human | Mammalia | 1 |
| Social | Tourism | Human | Mammalia | 1 |

*Table 2: Edge list of the Hawaiian S/EICAT(+) SENs, containing the starting node (from), end node (to), different link types (impact, mechanism), link weight (n), direction information for impact and mechanism (beneficial or deleterious), and status (observed or potential).*

| From | To | Impact | mechanism | n | Impact direction | Mechanism direction | Status |
| --- | --- | --- | --- | --- | --- | --- | --- |
| Cattle egret | Agriculture / aquaculture | Economic | Damage | 1 | Deleterious | Deleterious | Observed |
| Japanese white-eye | Agriculture / aquaculture | Economic | Damage | 1 | Deleterious | Deleterious | Observed |
| Rat species | Agriculture / aquaculture | Economic | Damage | 1 | Deleterious | Deleterious | Observed |
| Red-vented bulbul | Agriculture / aquaculture | Economic | Damage | 1 | Deleterious | Deleterious | Observed |
| Rose-ringed parakeet | Agriculture / aquaculture | Economic | Damage | 1 | Deleterious | Deleterious | Observed |
| Cattle egret | Airports | Health and safety | Risk of air strike at airports | 1 | Deleterious | Deleterious | Observed |
| Barn owl | Forest and grassland birds | Biodiversity | Predation | 1 | Deleterious | Deleterious | Observed |
| Feral cat | Forest and grassland birds | Biodiversity | Predation | 3 | Deleterious | Deleterious | Observed |
| Japanese white-eye | Forest and grassland birds | Biodiversity | Competition | 1 | Deleterious | Deleterious | Observed |
| Mongoose | Forest and grassland birds | Biodiversity | Predation | 2 | Deleterious | Deleterious | Observed |
| Plant communities | Forest and grassland birds | Biodiversity | Loss of native habitat | 6 | Beneficial | Deleterious | Observed |
| Rat species | Forest and grassland birds | Biodiversity | Predation | 13 | Deleterious | Deleterious | Observed |
| Veiled chameleon | Forest and grassland birds | Biodiversity | Predation | 1 | Deleterious | Deleterious | Potential |
| Forest and grassland birds | Hawaiian culture | Cultural | Loss of native species | 13 | Beneficial | Deleterious | Observed |
| Plant communities | Hawaiian culture | Cultural | Gain of native habitat | 5 | Beneficial | Beneficial | Observed |
| Plant communities | Hawaiian culture | Cultural | Loss of native habitat | 5 | Deleterious | Deleterious | Observed |
| Sea birds | Hawaiian culture | Cultural | Loss of native species | 6 | Beneficial | Deleterious | Observed |
| Wetland birds | Hawaiian culture | Cultural | Loss of native species | 5 | Beneficial | Deleterious | Observed |
| Red-vented bulbul | Monarch butterfly | Biodiversity | Predation | 1 | Deleterious | Deleterious | Observed |
| Red-whiskered bulbul | Monarch butterfly | Biodiversity | Predation | 1 | Deleterious | Deleterious | Observed |
| Feral cat | Pet owners | Emotional wellbeing | Ownership | 1 | Beneficial | Beneficial | Observed |
| Chukar | Plant communities | Biodiversity | Native seed dispersal | 1 | Beneficial | Beneficial | Observed |
| Chukar | Plant communities | Biodiversity | Gain of native habitat | 1 | Beneficial | Beneficial | Observed |
| Forest and grassland birds | Plant communities | Biodiversity | Native seed dispersal | 5 | Beneficial | Beneficial | Observed |
| Japanese white-eye | Plant communities | Biodiversity | Alien seed dispersal | 2 | Deleterious | Deleterious | Observed |
| Japanese white-eye | Plant communities | Biodiversity | Native seed dispersal | 2 | Beneficial | Beneficial | Observed |
| Japanese white-eye | Plant communities | Biodiversity | Gain of native habitat | 2 | Beneficial | Beneficial | Observed |
| Japanese white-eye | Plant communities | Biodiversity | Loss of native habitat | 2 | Deleterious | Deleterious | Observed |
| Rat species | Plant communities | Biodiversity | Predation | 5 | Deleterious | Deleterious | Observed |
| Red-billed leiothrix | Plant communities | Biodiversity | Alien seed dispersal | 2 | Deleterious | Deleterious | Observed |
| Red-billed leiothrix | Plant communities | Biodiversity | Native seed dispersal | 2 | Beneficial | Beneficial | Observed |
| Red-billed leiothrix | Plant communities | Biodiversity | Gain of native habitat | 2 | Beneficial | Beneficial | Observed |
| Red-billed leiothrix | Plant communities | Biodiversity | Loss of native habitat | 2 | Deleterious | Deleterious | Observed |
| Red-vented bulbul | Plant communities | Biodiversity | Alien seed dispersal | 2 | Deleterious | Deleterious | Observed |
| Red-vented bulbul | Plant communities | Biodiversity | Native seed dispersal | 2 | Beneficial | Beneficial | Observed |
| Red-vented bulbul | Plant communities | Biodiversity | Gain of native habitat | 2 | Beneficial | Beneficial | Observed |
| Red-vented bulbul | Plant communities | Biodiversity | Loss of native habitat | 2 | Deleterious | Deleterious | Observed |
| Red-whiskered bulbul | Plant communities | Biodiversity | Alien seed dispersal | 2 | Deleterious | Deleterious | Observed |
| Red-whiskered bulbul | Plant communities | Biodiversity | Native seed dispersal | 2 | Beneficial | Beneficial | Observed |
| Red-whiskered bulbul | Plant communities | Biodiversity | Gain of native habitat | 2 | Beneficial | Beneficial | Observed |
| Red-whiskered bulbul | Plant communities | Biodiversity | Loss of native habitat | 2 | Deleterious | Deleterious | Observed |
| Rose-ringed parakeet | Plant communities | Biodiversity | Alien seed dispersal | 2 | Deleterious | Deleterious | Observed |
| Rose-ringed parakeet | Plant communities | Biodiversity | Native seed dispersal | 2 | Beneficial | Beneficial | Observed |
| Rose-ringed parakeet | Plant communities | Biodiversity | Gain of native habitat | 2 | Beneficial | Beneficial | Observed |
| Rose-ringed parakeet | Plant communities | Biodiversity | Loss of native habitat | 2 | Deleterious | Deleterious | Observed |
| Forest and grassland birds | Recreation | Recreational potential | Loss of native species | 2 | Beneficial | Deleterious | Observed |
| Plant communities | Recreation | Recreational potential | Gain of native habitat | 5 | Beneficial | Beneficial | Observed |
| Plant communities | Recreation | Recreational potential | Loss of native habitat | 5 | Deleterious | Deleterious | Observed |
| Barn owl | Sea birds | Biodiversity | Predation | 5 | Deleterious | Deleterious | Observed |
| Cattle egret | Sea birds | Biodiversity | Predation | 2 | Deleterious | Deleterious | Observed |
| Common myna | Sea birds | Biodiversity | Predation | 1 | Deleterious | Deleterious | Observed |
| Feral cat | Sea birds | Biodiversity | Predation | 2 | Deleterious | Deleterious | Observed |
| Mongoose | Sea birds | Biodiversity | Predation | 2 | Deleterious | Deleterious | Observed |
| Plant communities | Sea birds | Biodiversity | Loss of native habitat | 6 | Beneficial | Deleterious | Observed |
| Rat species | Sea birds | Biodiversity | Predation | 2 | Deleterious | Deleterious | Observed |
| Rose-ringed parakeet | Tourism | Economic | Damage | 1 | Deleterious | Deleterious | Observed |
| Barn owl | Wetland birds | Biodiversity | Predation | 3 | Deleterious | Deleterious | Observed |
| Cattle egret | Wetland birds | Biodiversity | Predation | 4 | Deleterious | Deleterious | Observed |
| Feral cat | Wetland birds | Biodiversity | Predation | 4 | Deleterious | Deleterious | Observed |
| Mallard | Wetland birds | Biodiversity | Hybridisation | 1 | Deleterious | Deleterious | Observed |
| Mongoose | Wetland birds | Biodiversity | Predation | 1 | Deleterious | Deleterious | Observed |
| Plant communities | Wetland birds | Biodiversity | Loss of native habitat | 6 | Beneficial | Deleterious | Observed |
| Forest and grassland birds | Wildlife enthusiasts | Recreational potential | Loss of native species | 13 | Beneficial | Deleterious | Observed |
| Monarch butterfly | Wildlife enthusiasts | Recreational potential | Loss of native species | 1 | Beneficial | Deleterious | Observed |
| Sea birds | Wildlife enthusiasts | Recreational potential | Loss of native species | 6 | Beneficial | Deleterious | Observed |
| Wetland birds | Wildlife enthusiasts | Recreational potential | Loss of native species | 5 | Beneficial | Beneficial | Observed |
| Mongoose | Rat species | Biodiversity | Predation | 2 | Beneficial | Beneficial | Potential |
| Feral cat | Rat species | Biodiversity | Predation | 2 | Beneficial | Beneficial | Potential |
| Barn owl | Rat species | Biodiversity | Predation | 2 | Beneficial | Beneficial | Potential |

R packages

R version 4.3.2 (2023-10-31 ucrt) -- "Eye Holes"

Copyright (C) 2023 The R Foundation for Statistical Computing

Platform: x86_64-w64-mingw32/x64 (64-bit)

Csardi G, Nepusz T (2006). “The igraph software package for complex network research.” _InterJournal_, *Complex Systems*, 1695.

Csárdi G, Nepusz T, Traag V, Horvát Sz, Zanini F, Noom D, Müller K (2024). _igraph: Network Analysis and Visualization in R_. doi:10.5281/zenodo.7682609 <https://doi.org/10.5281/zenodo.7682609>, R package version 1.6.0,

Wickham H. ggplot2: Elegant Graphics for Data Analysis. Springer-Verlag New York, 2016.

Pedersen T (2022). _ggraph: An Implementation of Grammar of Graphics for Graphs and Networks_. R package version 2.1.0,

References

Anderson CJ, Brennan LA, Bukoski WP, Hess SC, Hilton CD, Shiels AB, Siers SR, Kluever BM, Klug PE. 2023. Evaluation of roost culling as a management strategy for reducing invasive rose-ringed parakeet (Psittacula krameri) populations. Biological Invasions 25: 1403–1419.

Anderson-Fung PO, Maly K. 2002. Hawaiian Ecosystems and Culture. Pages 177–205 in. Growing Plants for Hawaiian Lei; 85 Plants for Gardens, Conservation, and Business.

Cummings JL, Mason JR, Otis DL, Davis JE, Ohashi TJ. 1994. Evaluation of Methiocarb, Ziram, and Methyl Anthranilate as Bird Repellents Applied to Dendrobium Orchids. Wildlife Society Bulletin (1973-2006) 22: 633–638.

Evans T, Jeschke JM, Blackburn TM, Probert AF, Bacher S. 2020. Application of the Socio-Economic Impact Classification for Alien Taxa (SEICAT) to a global assessment of alien bird impacts. NeoBiota 62: 123–142.

Evans T, Kumschick S, Blackburn TM. 2016. Application of the Environmental Impact Classification for Alien Taxa (EICAT) to a global assessment of alien bird impacts. Diversity and Distributions 22: 919–931.

Fellows DP, Paton PWC. 1988. Behavioral response of cattle egrets to population control measures in Hawaii. Proceedings of the Vertebrate Pest Conference 13.

Kaushik M, Pejchar L, Crampton LH. 2018. Potential disruption of seed dispersal in the absence of a native Kauai thrush. PLOS ONE 13: e0191992.

Klug P, Bukoski W, Shiels A, Kluever B, Siers S. 2019. Rose-Ringed Parakeets. Wildlife Damage Management Technical Series.

Pyle RL, Pyle P. 2017. The Birds of the Hawaiian Islands: Occurrence, History, Distribution, and Status. B.P. Bishop Museum, Honolulu, HI, U.S.A. Version 2 (1 January 2017)

Raine AF, Vynne M, Driskill S. 2019. The impact of an introduced avian predator, the Barn Owl Tyto alba, on Hawaiian seabirds. Marine Ornithology 47: 33–38.

Rauzon MJ. 1978. Field observations from Kure Atoll, 1977. 'Elepaio 39:14.

Stimson J, Berman M. 1990. Predator induced colour polymorphism in Danaus plexippus L. (Lepidoptera: Nymphalidae) in Hawaii. Heredity 65: 401–406.

Kishinami KH. 2001. Birds. Pp. 21-27 in G.W. Staples and R.H. Cowie, eds., Hawaii's invasive species. B.P. Bishop Museum Press, Honolulu, HI.

Vizentin-Bugoni J, Tarwater CE, Foster JT, Drake DR, Gleditsch JM, Hruska AM, Kelley JP, Sperry JH. 2019. Structure, spatial dynamics, and stability of novel seed dispersal mutualistic networks in Hawaiʻi. Science 364: 78–82.

Global Invasive Species Database (GISD) 2024. Species profile Anas platyrhynchos. Available from:

https://www.iucngisd.org/gisd/species.php?sc=1241 [Accessed 21 March 2024]

Global Invasive Species Database (GISD) 2024. Species profile Alectoris chukar. Available from:

https://www.iucngisd.org/gisd/species.php?sc=1616 [Accessed 21 March 2024]

Global Invasive Species Database (GISD) 2024. Species profile Rattus exulans. Available from:

https://www.iucngisd.org/gisd/species.php?sc=170 [Accessed 21 March 2024]

Global Invasive Species Database (GISD) 2024. Species profile Rattus norvegicus. Available from:

https://www.iucngisd.org/gisd/species.php?sc=159 [Accessed 21 March 2024]

Global Invasive Species Database (GISD) 2015. Species profile Rattus rattus. Available from: https://www.iucngisd.org/gisd/species.php?sc=19 [Accessed 22 March 2024]

Global Invasive Species Database (GISD) 2024. Species profile Pycnonotus jocosus. Available from:

https://www.iucngisd.org/gisd/species.php?sc=1230 [Accessed 21 March 2024]

Global Invasive Species Database (GISD) 2024. Species profile Pycnonotus cafer. Available from:

https://www.iucngisd.org/gisd/species.php?sc=138 [Accessed 21 March 2024]

Global Invasive Species Database (GISD) 2024. Species profile Acridotheres tristis. Available from:

https://www.iucngisd.org/gisd/species.php?sc=108 [Accessed 21 March 2024]

Global Invasive Species Database (GISD) 2024. Species profile Herpestes javanicus. Available from:

https://www.iucngisd.org/gisd/species.php?sc=86 [Accessed 22 March 2024]

https://www.mmc.gov/priority-topics/species-of-concern/hawaiian-monk-seal/threats-to-hawaiian-monk-seals/#:~:text=Monk%20seals%20require%20terrestrial%20habitat,aquatic%20predators%20such%20as%20sharks.

https://dlnr.hawaii.gov/hisc/info/invasive-species-profiles/feral-cats/#:~:text=Feral%20cats%20have%20established%20populations,of%20Hawai'i's%20unique%20wildlife [Accessed 21 March 2024]

https://dlnr.hawaii.gov/hisc/info/invasive-species-profiles/mongoose/ [Accessed 21 March 2024]

https://dlnr.hawaii.gov/removerats/home/impacts-of-rodents-mongooses/#:~:text=The%20introduction%20of%20rodents%20and,sea%20turtle%20eggs%20and%20hatchlings [Accessed 21 March 2024]

https://kauaiseabirdproject.org/cultural-significance/ [Accessed 21 March 2024]

https://pacificbirds.org/2023/01/%CA%BBalae-%CA%BBula-the-bird-that-brought-fire/ [Accessed 21 March 2024]

https://www.nps.gov/hale/rare-winged-wonders-birds-of-haleakala.htm#:~:text=Native%20Hawaiian%20birds%20are%20significant,the%20'apapane%20or%20Hawaiian%20honeycreeper [Accessed 21 March 2024]

https://kauaiseabirdproject.org/the-threats/ [Accessed 21 March 2024]

https://onlinelibrary.wiley.com/doi/epdf/10.1111/j.1365-2486.2011.02464.x [Accessed 21 March 2024]

https://www.mauiforestbirds.org/cultural-significance/ [Accessed 21 March 2024]

https://www.reptileknowledge.com/reptile-pedia/which-bird-lead-people-to-the-hawaiian-islands [Accessed 21 March 2024]

https://abcbirds.org/noah-gomes-pilina/ [Accessed 21 March 2024]

https://ecos.fws.gov/ecp0/reports/ad-hoc-species-report accessed 17.05.24
